# Optimization of deep learning-based faster R-CNN network for vehicle detection

**DOI:** 10.1038/s41598-025-22828-z

**Published:** 2025-11-06

**Authors:** G Divya Deepak, Subraya Krishna Bhat

**Affiliations:** Department of Mechanical and Industrial Engineering, Manipal Institute of Technology, Manipal Academy of Higher Education, Manipal, 576104 Karnataka India

**Keywords:** Vehicle detection, CNN, Faster-RCNN, RCNN, Engineering, Mathematics and computing

## Abstract

Optimizing hyperparameters in object detection models is critical for enhancing performance, particularly in domain-specific tasks such as vehicle detection. This research systematically investigates the optimization of key hyperparameters for the Faster R-CNN model to maximize its efficiency in detecting vehicles. We evaluated the impact of various base CNN architectures (VGG-16, ResNet-50, Inceptionv3), solvers (sgdm, rmsprop, adam), learning rates (10^− 5^, 10^− 4^, 10^− 3^), and detection thresholds (0.1, 0.2, 0.3) on model performance. Our findings reveal that the optimal performance, achieving an average precision-recall (PR avg) value of 82%, was obtained using ResNet-50 with a learning rate of 10^− 5^ and a detection threshold of 0.1, employing the rmsprop solver across all learning rates and detection thresholds studied. The results demonstrate a clear trend wherein decreasing the learning rate from 10^− 3^ to 10^− 5^ steadily enhances network efficiency. Additionally, the choice of solver and detection threshold significantly influences the model’s performance. These insights emphasize the importance of meticulous hyperparameter tuning to improve the accuracy and reliability of object detection models. The proposed optimization methodology can be applied to various object detection tasks beyond vehicle detection, offering a framework for systematically enhancing model performance in diverse applications such as surveillance, autonomous driving, and traffic management systems.

## Introduction

Object detection is a critical task in computer vision, involving the identification and localization of objects within an image^1^. Object detection algorithms typically extract meaningful results using deep learning or machine learning models. Humans can locate and recognize objects of interest in a few moments as they look at an image or video. The objective of object detection is to duplicate this artificial intelligence. Recent years have seen substantial progress in this field, largely driven by advancements in Convolutional Neural Networks (CNNs) and deep learning^2^.

The evolution of CNN architectures has been a cornerstone of progress in object detection^3,4^. Notable architectures include VGG^5^, ResNet^6^, and more recently, EfficientNet^7^, etc. These networks have progressively increased the depth and complexity of CNNs while managing computational efficiency. For instance, ResNet introduced the concept of residual learning, which mitigates the vanishing gradient problem and allows for the training of very deep networks. EfficientNet, on the other hand, scales up the network’s depth, width, and resolution systematically, achieving a superior performance with fewer parameters.

Generally, deep learning methods of object detection employ CNNs such as single-shot detection (SSD)^8^, region-based CNN (R-CNN)^9^ and You only look once (YOLO)^10–12^. Region Proposal Networks (RPNs)^13^ have been instrumental in enhancing object detection frameworks. RPNs generate a set of candidate object bounding boxes (regions of interest) and have been integrated into models like Faster R-CNN^13^. This integration allows for end-to-end training and significantly speeds up the detection process compared to traditional methods such as selective search^14^. The introduction of RPNs marked a shift towards more efficient and accurate object detection models^15^.

In the R-CNN technique, there is a fusion of convolutional neural network features with rectangular region proposals. R-CNN is typically a detection algorithm that works in two stages. The first stage recognizes a subset of regions in an image that may contain an object. The second stage categorizes the object of each region. R-CNN-based object detectors are applied in various technological domains, including smart surveillance systems, autonomous driving, and facial recognition^16,17^.

Regarding vehicle detection, recent advancements using R-CNN models have significantly enhanced the ability to detect vehicles accurately and efficiently in various scenarios, including urban traffic, highways, and parking lots. The key developments revolve around improving the underlying R-CNN architecture and incorporating additional techniques to handle the unique challenges of vehicle detection^18,19^.

## Related work

As early as 2016, Fan et al.^20^ explored the performance improvement in Faster R-CNN network for vehicle detection tasks. They performed parameter tuning with respect to scaling of small sized objects to enhance the detection performance for vehicles with lesser pixel level presence in the images. Yang et al.^21^ used different deep neural networks such as VGG16, MobileNetv2, ResNet50, and ResNet101 to improve the performance of Faster R-CNN for vehicle detection from satellite based remote sensing images. A maximum accuracy of 83.2% in terms of the mean average precision (mAP) was achieved by using the ResNet101 model.

Recently, Abbas et al.^22^ developed an algorithm to determine traffic signal durations based on real-time vehicle density monitoring using Faster R-CNN network. They proposed the addition of another CNN for predicting region proposals for object detection which is then assigned to Faster R-CNN for detecting the object class. Furthermore, the model hyperparameters such as learning rate, image resizing, number of classes, feature extractor, batch size, optimizer, and number of epochs, were adjusted to enhance the predictive performance. The proposed approach achieved a maximum detection accuracy of 95.7%.

Bai et al.^23^ proposed an improvement by making changes in the ResNet model, which is a common backbone network for Faster R-CNN. A multi-layer feature combination process is proposed to retain both high-level and low-level features extracted by convolutional layers. Using the ResNet-101 backbone model, the authors achieved a maximum mAP of 83.2%, which was 1.7% higher than that for the unmodified Faster R-CNN. Chaudhuri et al.^24^ tackled the issues of occlusions, background clutters in traffic management systems by using adaptive background modeling along with the Faster R-CNN network for segmentation of vehicles.

Reddy et al.^25^ compared the different approaches of CNN-based object detection algorithms, including Faster R-CNN, YOLOv3, and Single Shot MultiBox Detector (SSD). Interestingly, Faster R-CNN, although ranking second in terms of the detection speed, demonstrated the lowest average loss among the three methods compared. Notably, the model hyperparameters such as the type of solver and batch size, were not addressed here. Hansen et al.^26^ demonstrated the superior accuracy of Faster R-CNN compared to SSD technique for unmanned aerial vehicle (UAV) based vehicle detection.

Researchers have attempted to modify the Faster R-CNN based network or couple it with other CNNs for improving vehicle detection performance in various contexts. Zhang et al.^27^ proposed a simple method of improving the detection accuracy of Faster R-CNN by modifying the Region Proposal Network (RPN) module in the network using filtering techniques to include the spatiotemporal information around the target objects (vehicles). Vijayakumar et al.^28^ compared two variations of Faster R-CNN by coupling it with Inceptionv2 and ResNet-101 models for improved object detection performance against benchmark datasets. Wadhwa et al.^29^ developed a real time system for automated detection of vehicles using aerial images by deploying a YOLOv8 and Faster R-CNN based object detection model. The lightweight nature of Faster R-CNN network and its ability to be deployed in embedded systems with applications in commercial humanoid robotics, smart factories and Artificial Intelligence of Things (AIoT) based devices^30–32^. Based on the literature review, the following research gaps are identified:

- While several works have enhanced the performance of Faster R-CNN through architectural modifications (e.g., ResNet improvements by Bai et al.^23^, multi-CNN approaches by Abbas et al.^22^, and RPN module refinement by Zhang et al.^27^, there is limited focus on systematically optimizing key hyperparameters, such as the solver, detection threshold, and learning rate, to achieve task-specific performance improvements^33–36^.
- Previous works (e.g., Reddy et al.^25^ have compared Faster R-CNN with other object detection algorithms but did not systematically tune critical hyperparameters.
- Works such as Yang et al.^21^ have explored various base CNNs like VGG-16 and ResNet-101 for vehicle detection. However, these studies do not extend to comparing newer architectures, such as Inceptionv3, in the context of Faster R-CNN.
- Prior studies (e.g., Chaudhuri et al.^24^, Hansen et al.^26^ have shown Faster R-CNN’s superior performance in specific scenarios, such as UAV-based detection or occluded environments, but lack a systematic investigation of the interplay between hyperparameters and detection tasks like vehicle detection.

The present work aims to mitigate the identified research gaps by conducting a comprehensive optimization of key hyperparameters in Faster R-CNN, including:

- Base CNN Architectures: VGG-16, ResNet-50, and Inceptionv3 are evaluated to identify the most suitable backbone for vehicle detection.
- Solvers: Comparisons among stochastic gradient descent with momentum (sgdm), root mean square propagation (rmsprop), and adaptive moment estimation (adam) solvers.
- Detection Thresholds: Fine-tuning of thresholds (0.3, 0.2, and 0.1) to ensure optimal sensitivity for detecting vehicles with varying pixel sizes.
- Learning Rates: Learning rates (10^− 3^, 10^− 4^, 10^− 5^) are adjusted for stable and efficient training.

This research thus provides a detailed framework for optimizing Faster R-CNN for deployment in real-world vehicle detection applications, addressing the lightweight and real-time requirements mentioned by Wadhwa et al.^29^ and Abbas et al.^22^.

## Methodology

The methodology for object detection using CNN includes the following basic steps. The first step is to recognize regions contained in an image that might contain an object. These regions are named as region proposals. Secondly, CNN extracts the relevant features from the region proposals. Finally, a classifier is used to classify the objects and thereby identify the required objects to be identified by means of the extracted features.

Beginning with a background on the different variants of R-CNN networks, this section introduces the working principle of Faster R-CNN and Region Proposal Network (RPN). Further, the dataset used to train the models, and the procedures carried out to augment the dataset are discussed. Next the CNN network architectures investigated in the present work are introduced. Finally, the methodology proposed for optimizing the hyperparameters of the Faster R-CNN model is presented. The models employed in the present work are developed using the Computer Vision Toolbox™ of MATLAB 2023b (MathWorks Inc.).

### Faster-RCNN

The evolution of R-CNN (Region-based Convolutional Neural Networks) and its variants represent a significant milestone in the field of object detection, culminating in models that are both more accurate and computationally efficient. The discussion on Faster R-CNN is but incomplete without exploring its predecessors – R-CNN and Fast R-CNN.

**Fig. 1:**
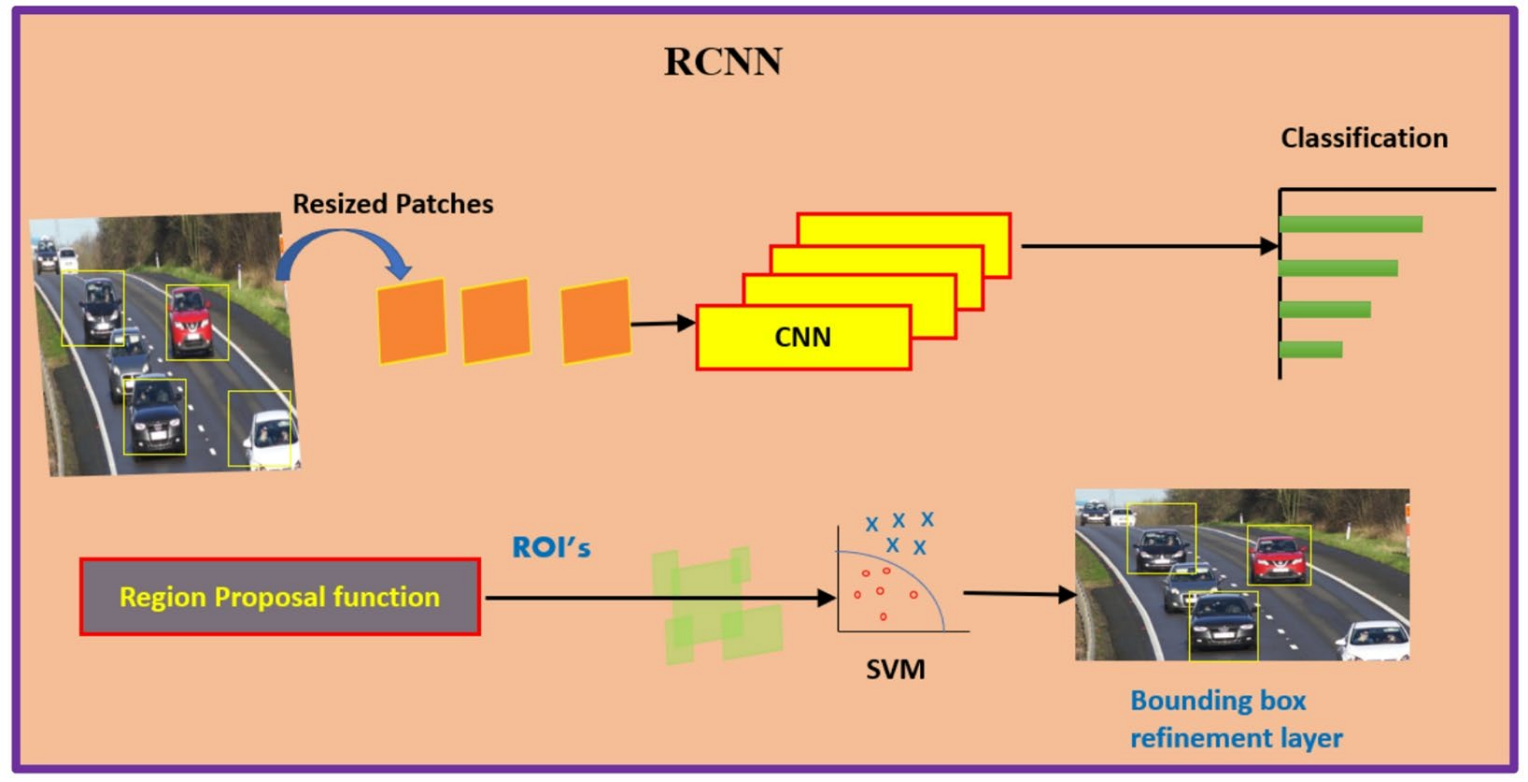
R-CNN object detection algorithm.

Introduced by Girshick et al.^9^ in 2014, the original R-CNN was groundbreaking for its time. It employed a selective search algorithm to generate region proposals, which were then classified using CNNs. Each of the region proposal was independently processed, which made the method computationally expensive and slow, but it set a new standard for object detection accuracy. The R-CNN detector first produces region proposals by means of an algorithm such as Edge Boxes^37^. The proposal regions are cropped from the image and resized. Then, CNN classifies the resized and cropped regions. Further, the region proposal bounding boxes are refined by applying support vector machine (SVM) which is pretrained from CNN features. Figure 1 shows the schematic depiction of the working mechanism of R-CNN.

**Fig. 2:**
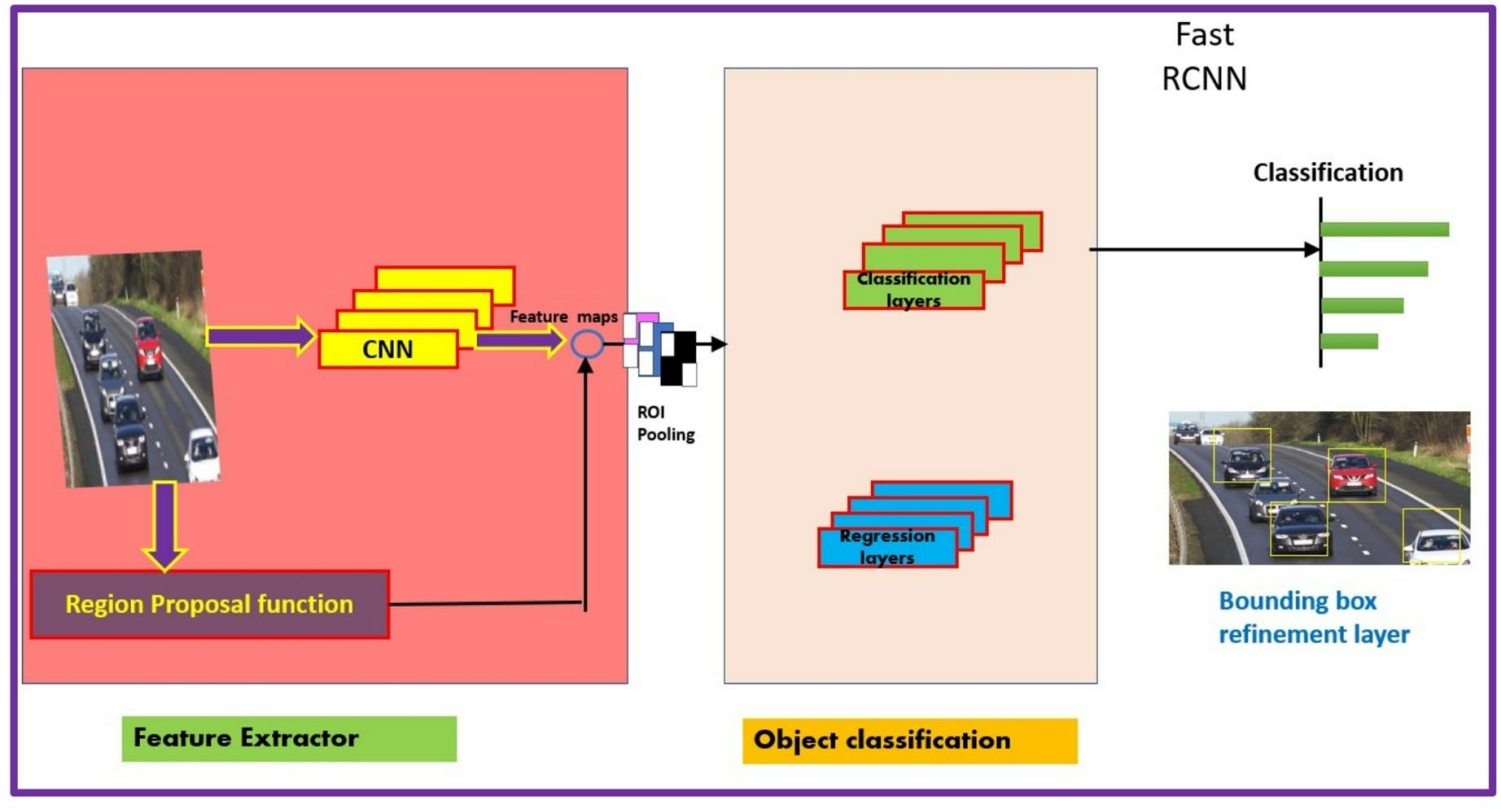
Fast R-CNN object detection algorithm.

As in the case of R-CNN, the Fast R-CNN detector introduced by Girshick^38^ in 2015 employs the Edge Boxes algorithm to generate region proposals. However, Fast R-CNN improved upon R-CNN by integrating the region proposal and classification stages into a single network. It introduced the Region of Interest (RoI) pooling layer (refer Fig. 2), which allowed for the extraction of fixed-size feature maps from each proposed region. This integration enabled shared computation, significantly speeding up the process and reducing the redundancy inherent in R-CNN.

**Fig. 3:**
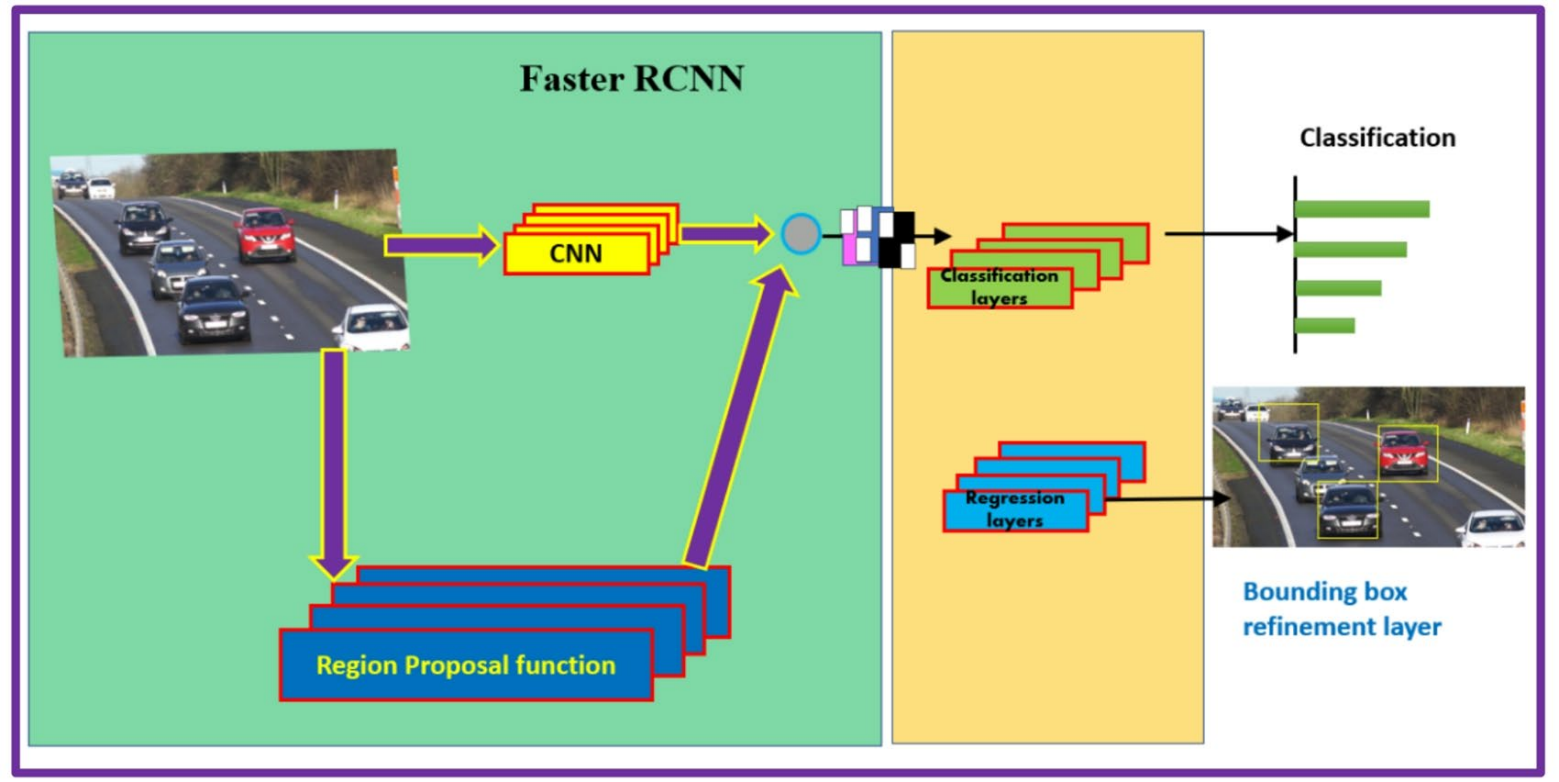
Faster R-CNN object detection algorithm.

Faster R-CNN, developed by Ren et al.^13^ in 2015, further streamlined the process by introducing the Region Proposal Network (RPN). The RPN generates region proposals directly from the feature maps produced by the convolutional layers, eliminating the need for an external region proposal method like selective search or Edge Boxes method (refer Fig. 3). The RPN applies anchor boxes for object detection, and thus creating region proposals in the network is better and faster according to the given data. This innovation allowed for near real-time object detection and significantly improved efficiency without compromising accuracy.

**Fig. 4:**
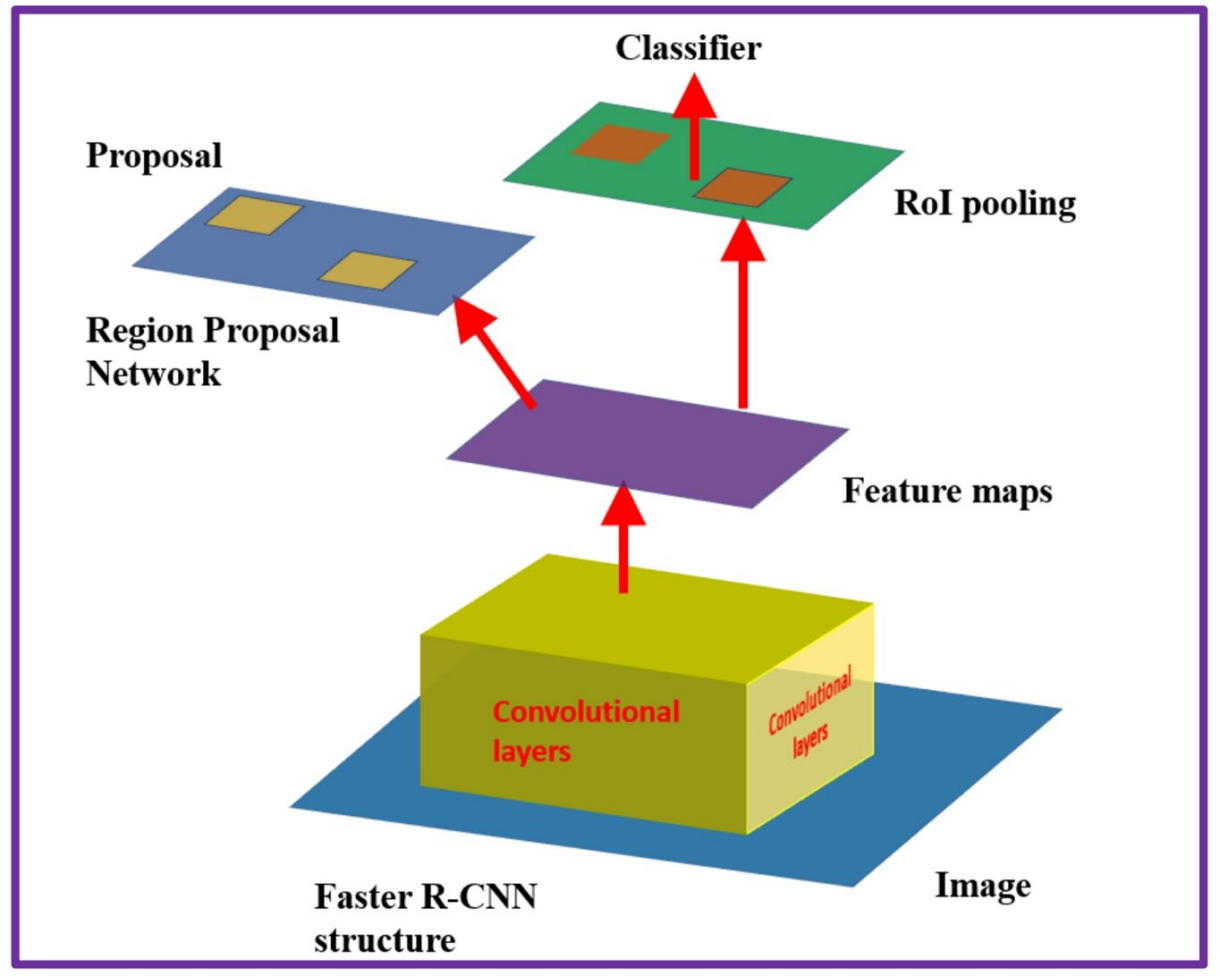
The Faster R-CNN detector structure^13^.

The Faster R-CNN detector comprises of two modules. The first module is the Fully Convolutional Network (FCN) applied to generate a region proposal network (RPN), and the second module is the Fast R-CNN implemented as a detector based on the proposal region of the first module. The second module (RPN) helps Faster R-CNN to identify the region of interest (ROI), accelerating the computation process. The configuration of the Faster R-CNN model is shown in Fig. 4.

### Region proposal network

**Fig. 5:**
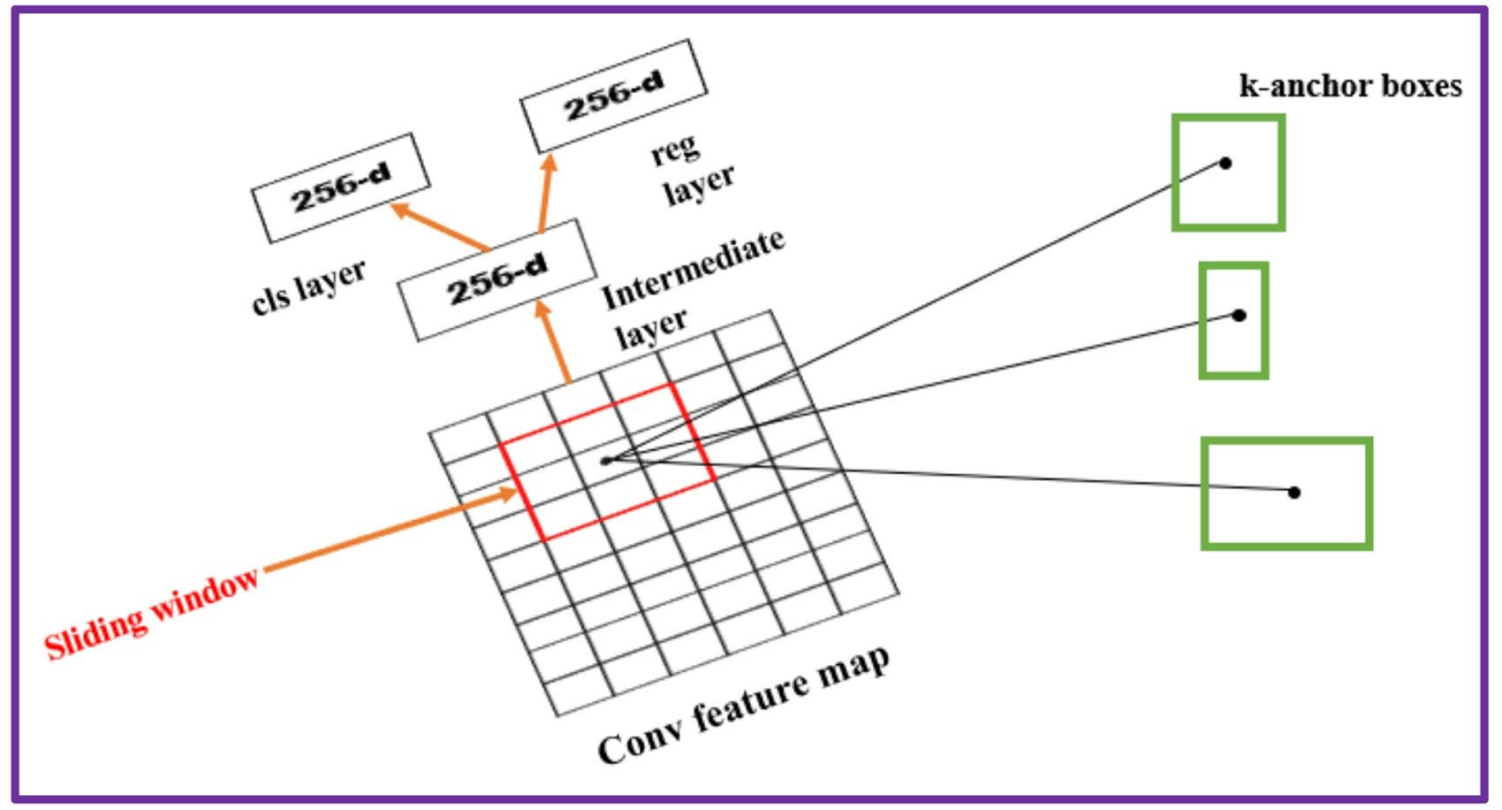
The region proposal network architecture^13^.

Region Proposal Network (RPN) obtains the image as input and generates a set of squares with the proposal object (location of object), each containing an object score^13^. RPN maps the last layer of CNN using a 3$\:\times\:$3 sliding window to a reduced dimension to obtain the feature map. The role of RPN is to generate several ROI which have a greater probability of covering an object. The RPN architecture is depicted schematically in Fig. 5.

The final feature map contains two layers, which are the *cls* layer and the *reg* layers. The central point of sliding windows is the anchor. To enable diverse object sizes, anchor box dimensions vary from, 1$\:\times\:$2, 1$\:\times\:$1 or 2$\:\times\:$1. The *cls* layer carries 2000 estimated values of possible objects for every proposal, where total sum of the possible locations of objects is indicated by *k*. Because for sliding windows a 3$\:\times\:$3 matrix is used, the total likelihood is 9 pixels. The *reg* layer carries 4000 coordinates from the location of the *k* box. The 4 coordinates are in the middle box (*x*, *y*), width (*w*)/width, and height(*h*)/height. A non-maximum suppression is done to reduce the number of anchor boxes, where intersecting anchor boxes would be deleted if contains lower value of intersection over union. The limits set for the union is more than 0.7 (positive objects) and lesser than 0.3 for (negative/background). The calculation of Intersection over Union is as follows:

1 $$\:IoU=\frac{Anchor\:box\cap\:Ground\:Truth\:box}{Anchor\:box\:\cup\:Ground\:Truth\:box}$$

The RPN is an algorithm that requires training, so RPN has a loss function, shown in (2)^24^.

2 $$\:L\left({\{p}_{i}\right\},{\{t}_{i}\left\}\right)=\frac{1}{{N}_{cls}}\sum\:_{i}{L}_{cls}\left({p}_{i},{p}_{i}^{*}\right)+\lambda\:\frac{1}{{N}_{reg}}\sum\:_{i}{p}_{i}*{L}_{reg}({t}_{i},{t}_{i}^{*})$$

where, $\:{p}_{i}^{*}$ is the Ground truth label and $\:{t}_{i}^{*}$ is the corresponding Ground truth coordinate. *p*_*i*_ and *t*_*i*_ are the object possibility and the 4*k* anchor coordinate, respectively. *L*_*cls*_ represents the classification loss (log loss) and *L*_*reg*_ indicates the regression loss (smooth L_1_ loss). Further, *N*_*cls*_ and *N*_*reg*_ are the classification normalization and regression normalizations, respectively. Also, λ is a balancing parameter to balance between the *cls* and *reg* layers. The calculation of anchor coordinate is shown in Eqs. (3-10)^25^.

3 $$\:{t}_{x}=\frac{(x-{x}_{a})}{{w}_{a}}$$

4 $$\:{t}_{y}=\frac{(y-{y}_{a})}{{h}_{a}}$$

5 $$\:{t}_{w}=log\frac{w}{{w}_{a}}$$

6 $$\:{t}_{h}=log\frac{h}{{h}_{a}}$$

7 $$\:{t}_{x}^{*}=\frac{({x}^{*}-{x}_{a})}{{w}_{a}}$$

8 $$\:{t}_{y}^{*}=\frac{({y}^{*}-{y}_{a})}{{w}_{a}}$$

9 $$\:{t}_{w}^{*}=log\frac{{w}^{*}}{{w}_{a}}$$

10 $$\:{t}_{h}^{*}=log\frac{{h}^{*}}{{h}_{a}}$$

Wherein, *y* and *x* are the prediction box coordinates of y-axis and x-axis, respectively. The width and height of prediction box are represented by *w* and *h*, respectively. The anchor box is indicated by *a*. The loss function classifier and loss function bounding box calculations are shown in Eq. (11) and Eq. (12), respectively.

11 $$\:{L}_{cls}({t}_{i},{{t}_{i}}^{*})={{-(p}_{i}}^{*}\text{log}\left({p}_{i}\right)+(1-{{p}_{i}}^{*})\text{l}\text{o}\text{g}(1-{p}_{i})),$$

12 $$\:{L}_{reg}\left({t}_{i},{{t}_{i}}^{*}\right)={\varSigma\:}_{i\:\left\{x,y,w,h\right\}}{smooth}_{L1}\left({t}_{i}-{t}_{i}^{*}\right).$$

Where,

13 $$\:{smooth}_{L1\:}\left({t}_{i}-{t}_{{i}^{*}}\right)=\left\{\begin{array}{c}{0.5x}^{2}\:\:\:\:\:\:\:\:\:\:\:if\:\left|{t}_{i}-{t}_{{i}^{*}}\right|<1\\\:\left|x\right|-0.5\:\:\:\:\:\:\:\:\:\:\:\:\:\:\:\:\:\:\:\:\:\:\:\:other\end{array}\right..$$

**Fig. 6:**
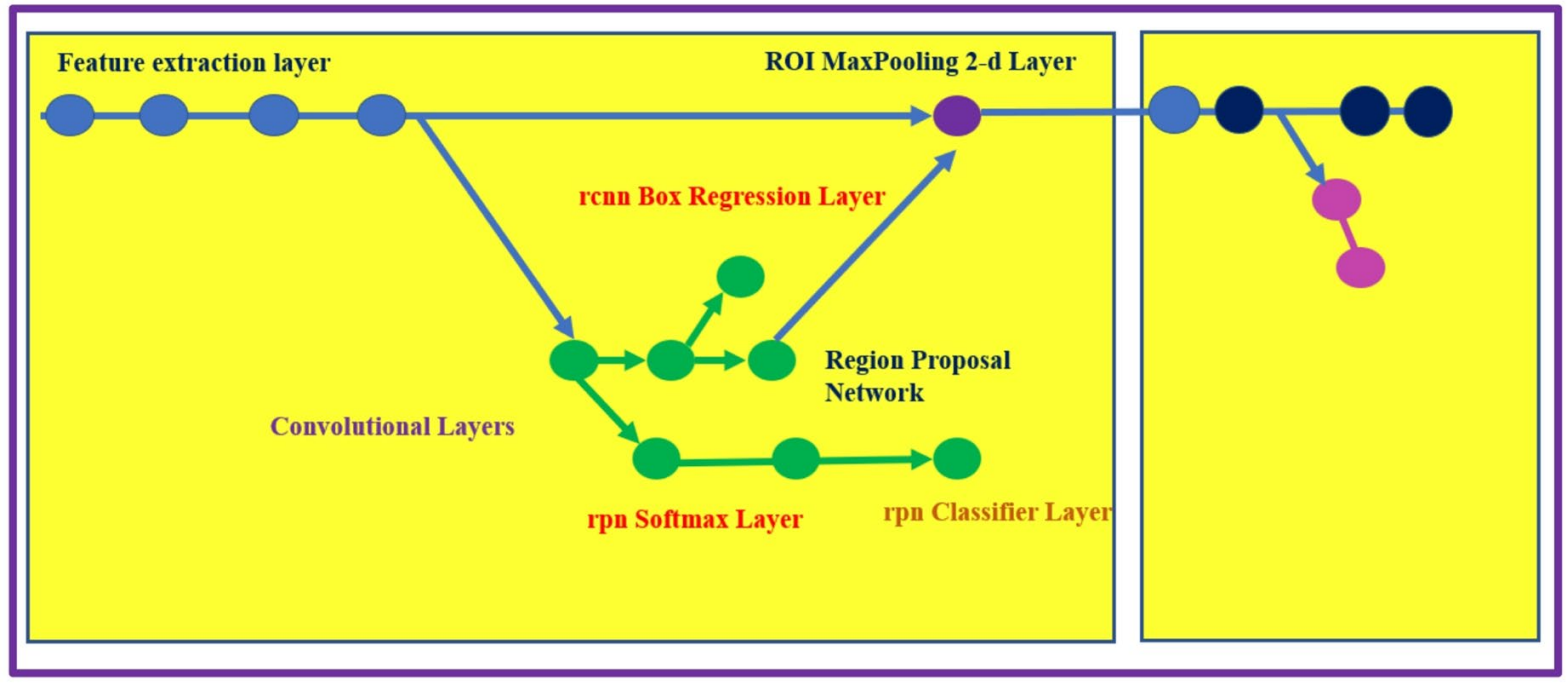
The Faster R-CNN network showing the different layers^13^.

The Faster R-CNN network structure with the different feature extraction layers and MaxPooling layers is depicted in Fig. 6.

### Dataset

The Caltech Cars 1999 dataset collated by Weber and Perona^39^ from Caltech is used in this study to train the network. It consists of 126 JPEG images of cars from the rear-view side with a size of 896$\:\times\:$592 pixels. Approximate scale normalization has been conducted. The pictures were taken in the Caltech parking lot. In the current work, 80% of the dataset is used for training the Faster-RCNN network and the remaining 20% is used for testing. Also, the shuffle command is used to return a datastore object containing a random ordering of the data from the original dataset.

### Data augmentation

Data augmentation is used to improve network accuracy by randomly transforming the original data during training. By using data augmentation, additional variety is incorporated in the training data without actually having to increase the number of labeled training samples. The *transform* function of MATLAB is utilized to augment the training data by randomly flipping the associated box labels and images horizontally. After data augmentation 25% of dataset images were added to the original dataset. Hence the total number of images after data augmentation was 370 which included 296 training images (80% of dataset) and 74 test images (20% of dataset). The data augmentation methods result in enhanced accuracy and improved model generalization. Data augmentation techniques are applied to produce a robust model and avoid evaluation bias.

### Label training data for deep learning

The Image Labeler™ app in MATLAB has been employed to label pixels and export label data required for training. This app is useful for rectangular regions of interest (ROIs) intended for object detection. The scene labels are also employed for the classification of images and pixels for semantic segmentation.

### Region proposal network anchor boxes for object detection

**Fig. 7:**
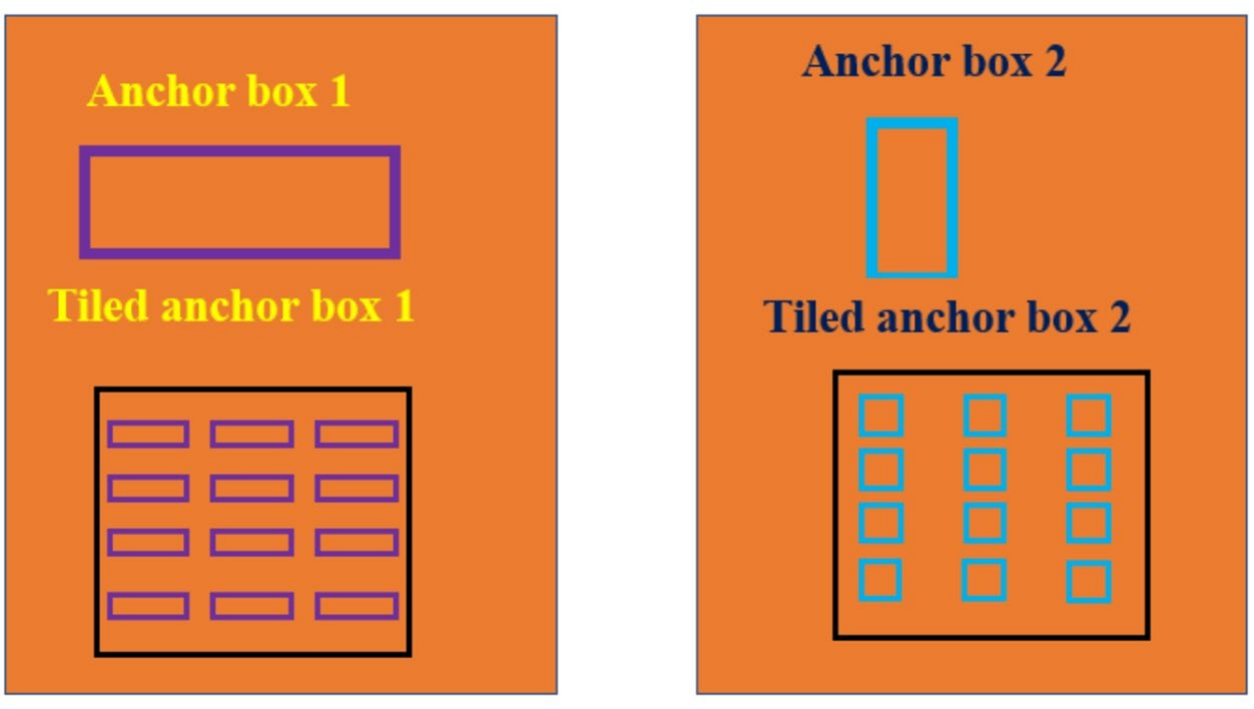
Anchor boxes for object detection.

Deep learning neural network-based object detection provides accurate and fast techniques to predict the size and location of object in an image. Generally, the network returns valid objects in regular time intervals irrespective of the scale of objects. The implementation of anchor box enhances the efficiency and speed of detection in the deep learning framework. The network computes the probability and other characteristics such as intersection over union (IoU), background and offsets for each tiled anchor box (refer Fig. 7). The predictions are utilized to refine each anchor box. The network only predicts the refinements and probabilities corresponding to each tiled anchor box and cannot predict the bounding boxes directly. For every anchor box, the network returns a unique set of predictions. The object detections in each class are represented by the final feature map. The anchor box deployment helps the network to detect overlapping objects, multiple objects, and objects of different scales.

**Fig. 8:**
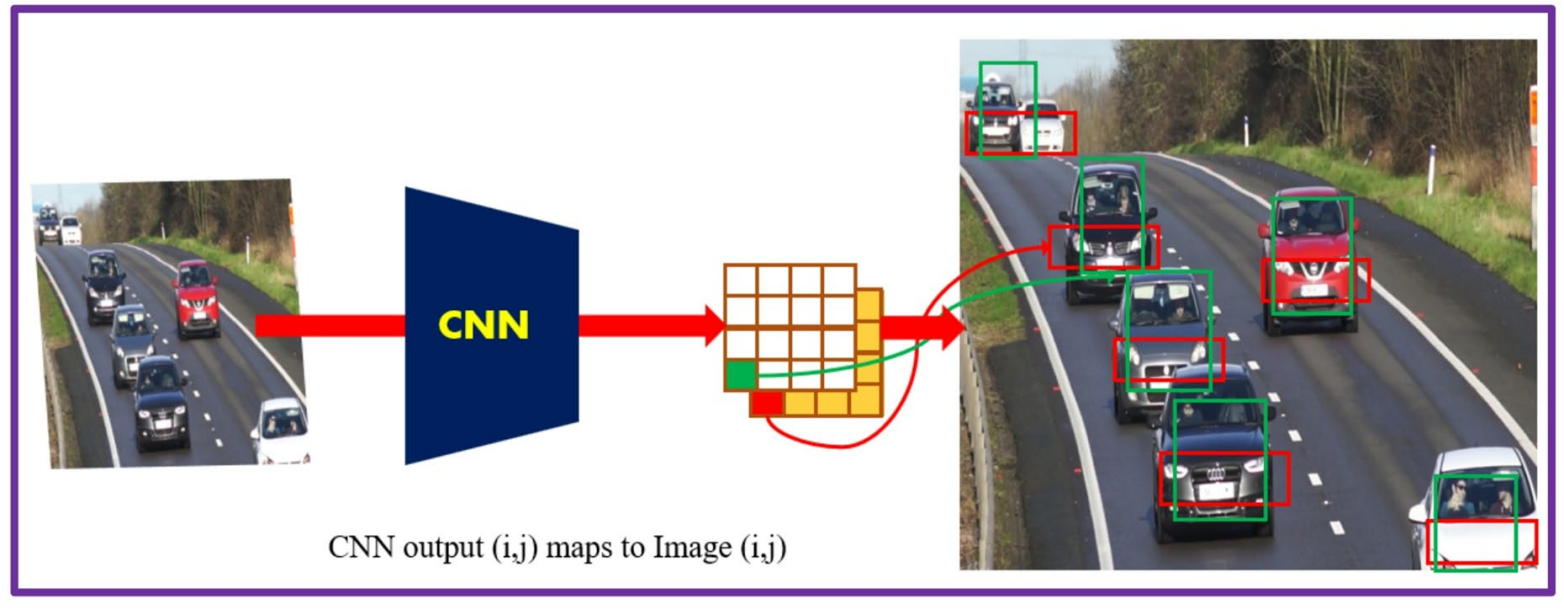
Anchor boxes for prediction of a particular class.

**Fig. 9:**
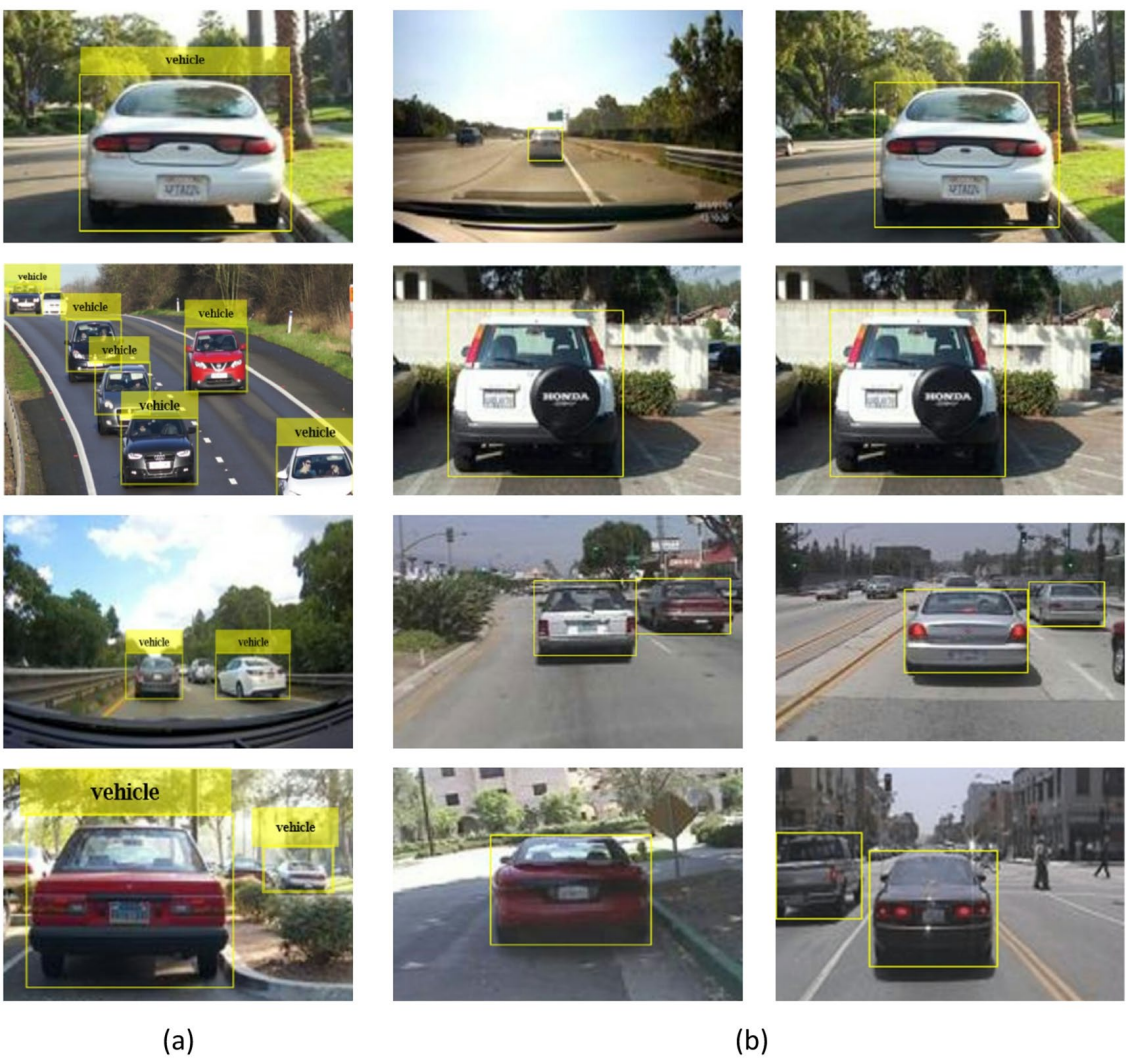
Sample images from the dataset with (**a**, **b**) anchor boxes and (**b**) labels for vehicle detection using Faster R-CNN.

The anchor box position is computed by mapping the location of network output back to the input image. The process is repeated for each network output. The results generate a group of tiled anchor boxes across the image. Each anchor depicts a particular prediction belonging to a class. For e.g., Fig. 8 shows two anchor boxes that generate two predictions per location for an image. Multiprocessing can be applied to enable the network to detect objects of varying sizes. Figure 9 presents some sample images from the dataset with anchor boxes for vehicle detection.

### Proposed optimization methodology for faster R-CNN

**Fig. 10:**
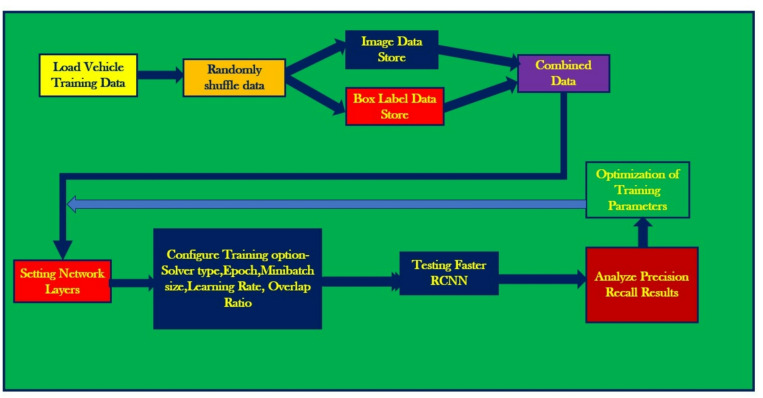
Workflow of Faster R-CNN object detection algorithm for vehicle detection along with the procedure for optimization of hyperparameters (training parameters).

The objection detection using Faster R-CNN employs the workflow depicted in Fig. 10. It involves loading the vehicle training data and then shuffling it randomly to avoid any bias occurring during the train/test data splitting. Further, this shuffled vehicle data is used to create an image data store (*imds*) and box label data store (*blds*) which are then used to create the combined data store (*cds*). This is followed by the process of training parameters optimization, which includes setting the network layers and configuring the training options (solver type, epoch, mini batch size, learning rate, overlap ratio). The configured Faster R-CNN is trained and tested with the corresponding image data to evaluate the precision-recall results. This process is repeated for different combinations of training options (hyperparameters) – base network (VGG-16, ResNet-50, Inceptionv3), solver (sgdm, rmsprop, adam), detection threshold (0.3, 0.2 and 0.1) and learning rates (10^− 3^, 10^− 4^ and 10^− 5^) to assess their influence on the vehicle detection performance. The detection threshold is kept constant at 0.3^27^. To mitigate the effect of sampling biases, 5-fold cross-validation was performed by splitting the dataset into 5 subsets, training on one subset and testing on other subsets during each iteration.

### Network architectures

In this research work, three Faster R-CNN based networks using VGG-16, ResNet-50 and Inceptionv3 have been employed for vehicle detection. These architectures were selected to represent three distinct families of CNN backbones that differ in design philosophy, computational complexity, and feature extraction capabilities.

- VGG-16: Proposed by Simonyan and Zisserman^4^, VGG-16 emphasizes architectural simplicity with a deep stack of small 3 × 3 convolutional filters. While computationally more expensive, it provides a strong baseline with effective feature representation, making it suitable for controlled conditions.
- ResNet-50: Introduced by He et al.^6^, ResNet-50 incorporates residual connections that mitigate the vanishing gradient problem, enabling stable training of deeper networks. This architecture provides robustness in detecting vehicles under challenging conditions such as occlusion, shadows, and varying backgrounds due to its ability to learn hierarchical features effectively.
- Inceptionv3: Developed by Szegedy et al.^40^, Inceptionv3 focuses on computational efficiency by factorizing convolutions and incorporating multi-scale feature extraction within each inception block. This makes it particularly well-suited for detecting vehicles of varying sizes and aspect ratios under diverse environmental conditions (e.g., lighting, weather).

Thus, the three networks were chosen to provide a balanced evaluation across baseline (VGG-16), deep residual (ResNet-50), and multi-scale efficient (Inceptionv3) approaches, ensuring that the optimization framework captures variations in both computational cost and detection performance. The network type and feature extraction layers in each of the networks are shown in Table 1.

**Table 1:** Details of faster R-CNN networks used in the present work.

Network Type	Feature Extraction Layer	Description
VGG-16	*relu5_3*	Last max pooling layer is replaced by ROI max pooling layer
ResNet-50	*activation_40_relu*	ROI pooling layer is inserted after the feature extraction layer.
Inceptionv3	*mixed7*	ROI pooling layer is inserted after the feature extraction layer.

**Fig. 11:**
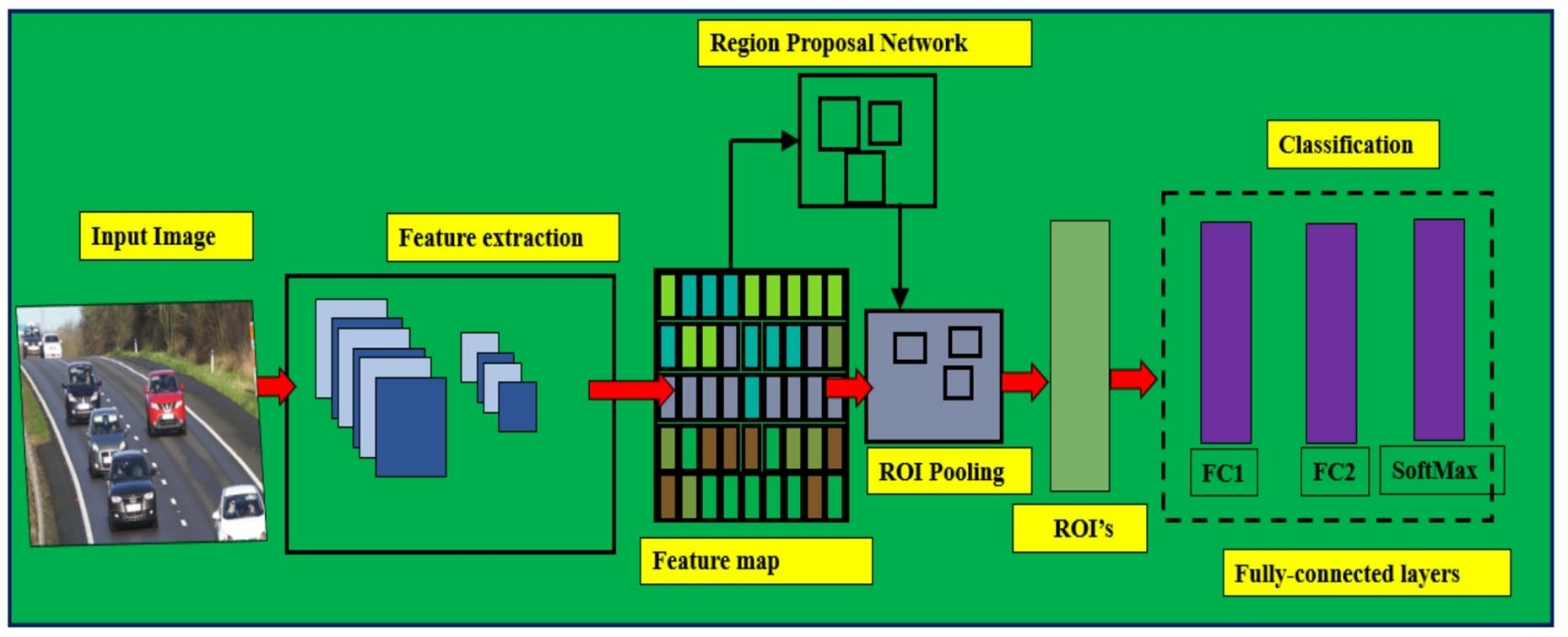
VGG-16 network architecture.

The first CNN considered in this study is VGG-16, which was proposed by Simoyan and Zisserman^40^ in 2014. The architecture is characterized by its simplicity, using a stack of 3 × 3 convolutional layers and 2 × 2 max-pooling layers, followed by fully connected layers. With 16 weight layers, VGG-16 emphasizes depth to enhance feature representation. The VGG16 network took second place in the 2014 ImageNet Large Scale Visual Recognition Challenge Competition (ILSVRC). Figure 11 shows the network architecture of VGG-16. The feature extraction layer employed in VGG-16 is *relu5_3*.

**Fig. 12:**
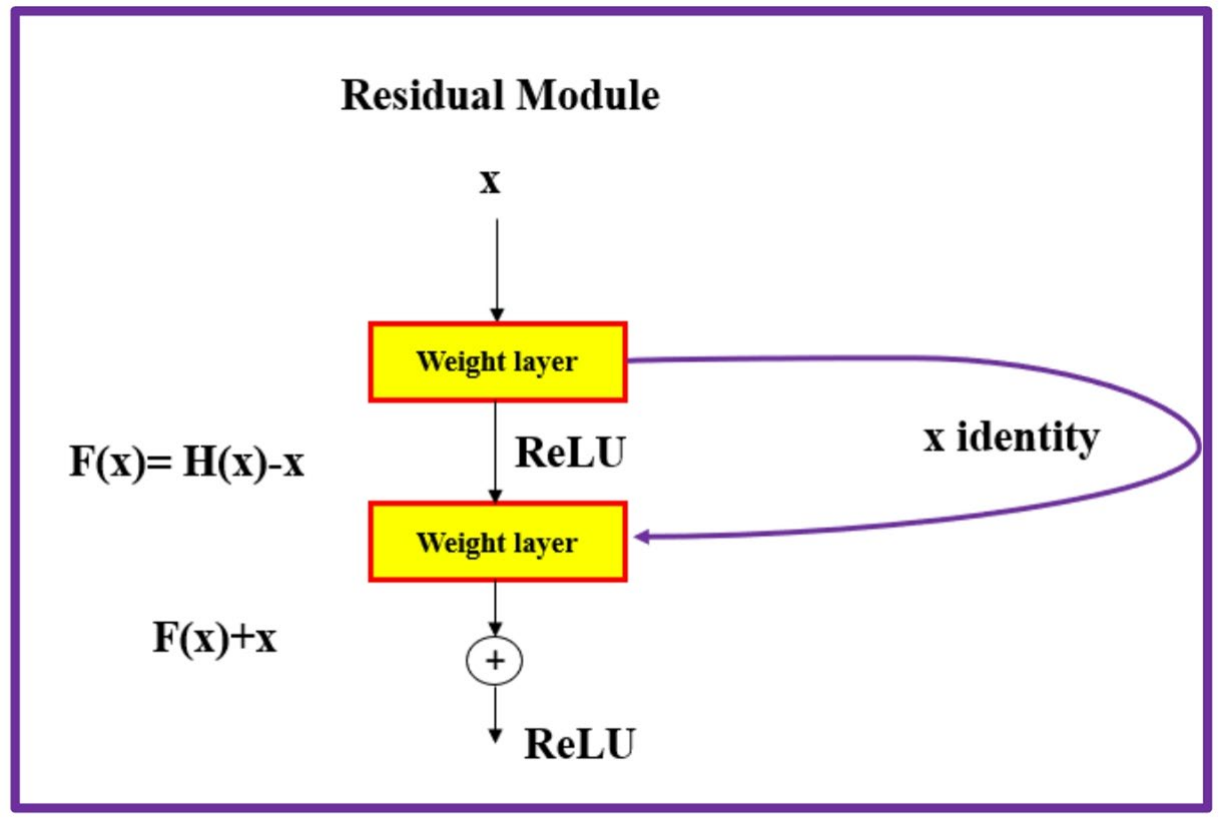
ResNet with residual module.

**Fig. 13:**
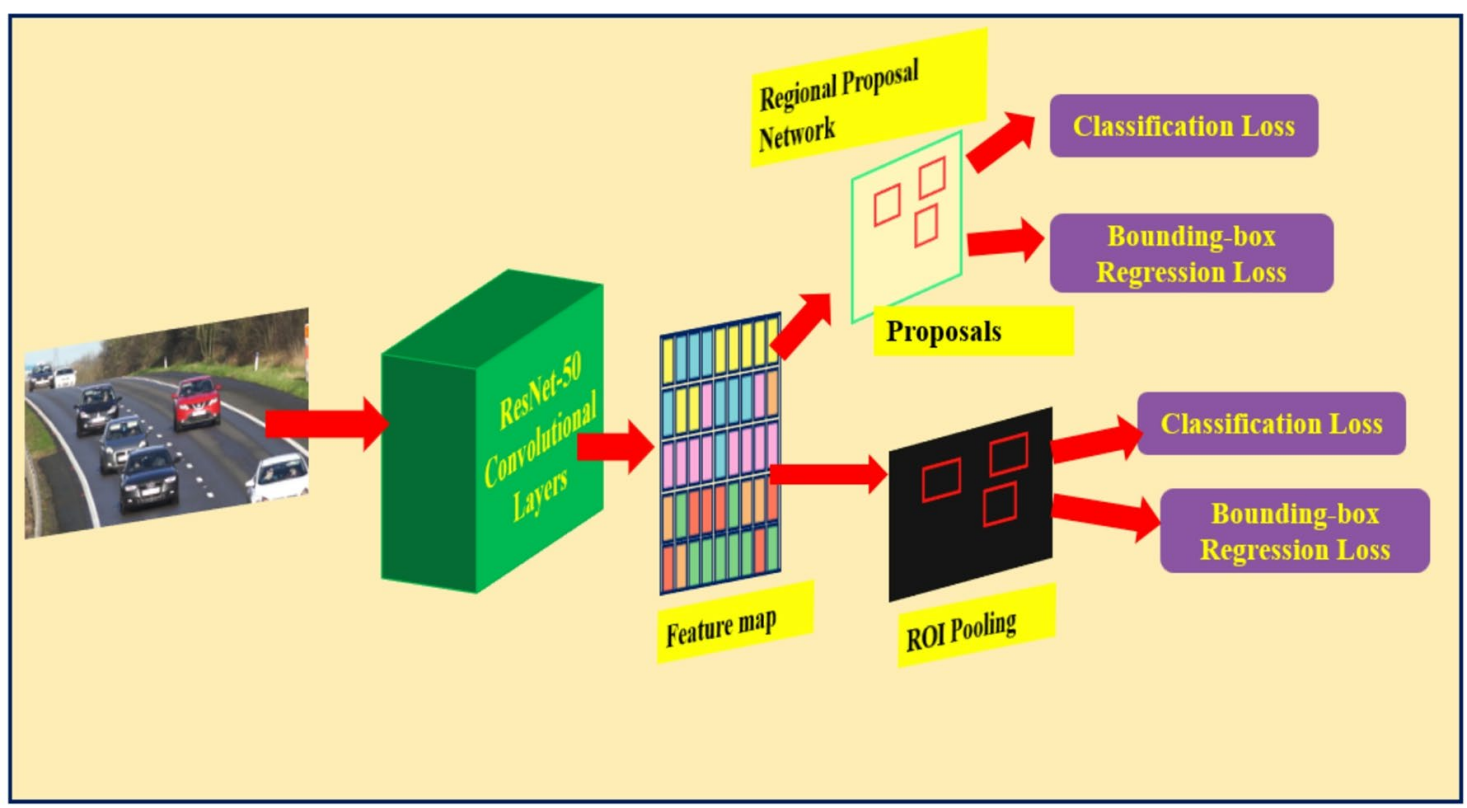
Faster R-CNN network based on ResNet-50 for vehicle detection.

The second network considered here in this study is ResNet-50. During the course of deep network formation, the value of gradient becomes saturated with small values or attains large value, thus causing a vanishing gradient issue that slows the learning effect. ResNet^6^ makes an addition of identity shortcut connection to the conventional neural network to extract the learning effect of the deep network. As the shortcut connection interlinks the input to the output directly without parameters, only computation is added. Optimization of deep networks can be done due to this identity shortcut and accuracy improved due to the possibility of developing efficient deeper networks. The feature extraction layer employed in ResNet-50 is *activation_40_relu*. This particular feature extraction layer generates feature map outputs that are down sampled by a factor of 16, which provides a good trade-off between strength of extracted features and spatial resolution, as features extracted down the network encode important features of image at the cost of spatial resolution. Figure 12 shows the block of residual learning that composes ResNet^6^. Previously, H(*x*) was learned, but residual learning acquires H(*x*) – *x*. The improved method in ResNet way picks up in a direction where H(*x*) – *x* must be 0, thus increasing the likelihood of detecting movements with small inputs effortlessly. Figure 13 presents the network architecture of Faster R-CNN object detector based on ResNet-50.

**Fig. 14:**
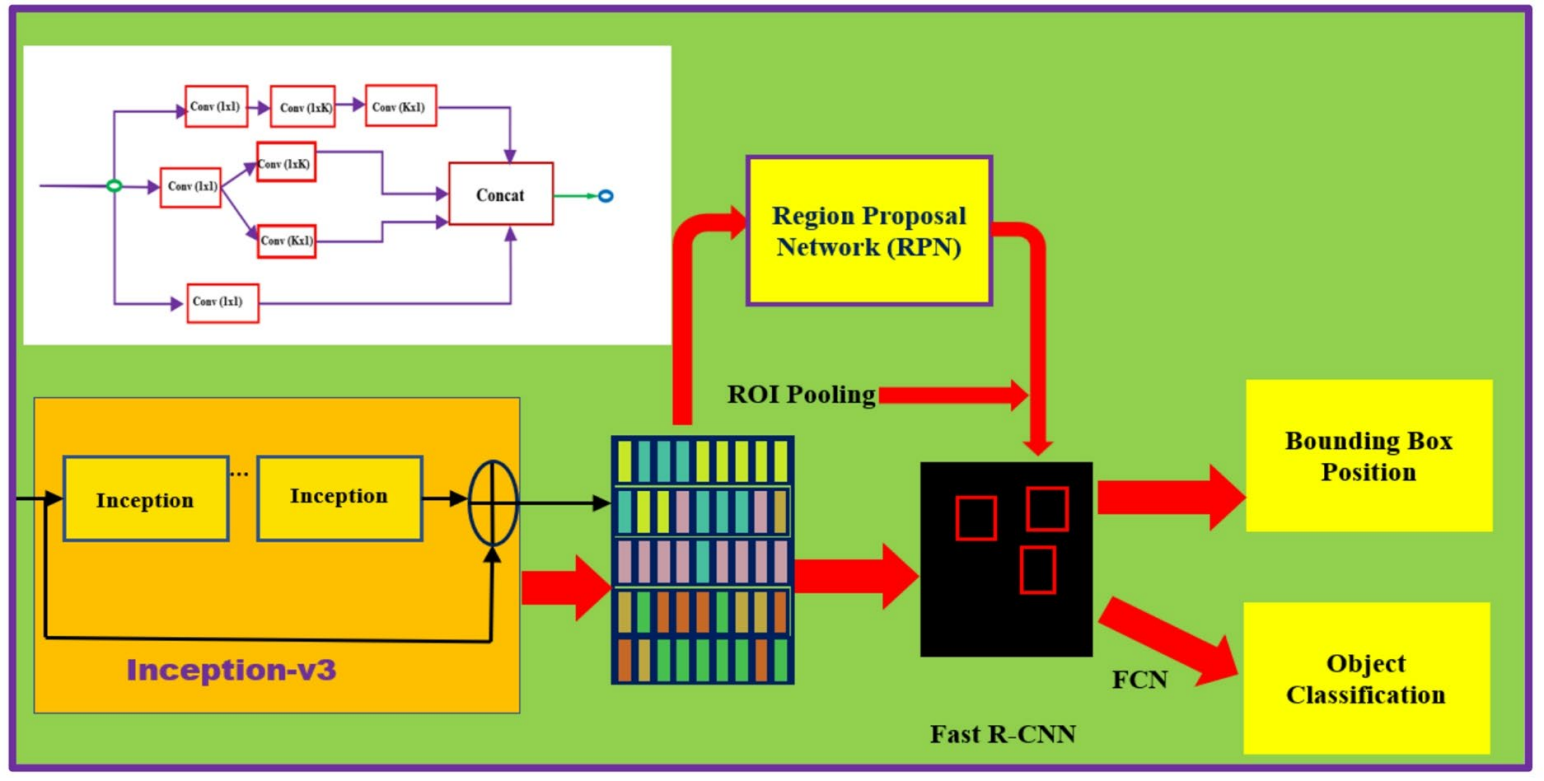
Faster R-CNN based on Inceptionv3 for vehicle detection.

The third CNN considered here is Inceptionv3. Inceptionv3, introduced by Szegedy et al.^40^ in 2015, is a significant advancement in CNN architectures, improving upon previous versions with greater efficiency and accuracy. It utilizes factorized convolutions and aggressive regularization, enabling deeper networks without increasing computational cost. The architecture also includes the use of auxiliary classifiers and batch normalization for faster training and better performance^41^. These enhancements allow Inceptionv3 to achieve state-of-the-art results on various image classification tasks, balancing depth, width, and resolution for optimal performance. The feature extraction layer employed in Inception-v3 is *mixed7*. Figure 14 presents the network architecture of Faster R-CNN object detector based on Inceptionv3.

## Results and discussion

In this research work, optimization of network parameters has been carried out for Faster R-CNN network based on VGG-16, ResNet-50 and Inception-v3. The performance of the Faster R-CNN based networks has been computed using the Precision-Recall (PR) curve and confidence scores. The average PR value elaborates on the capacity of the detector to make correct classifications (precision) and the capability of the detector to find all appropriate objects (recall). Each of these three networks are then analyzed at three levels of learning rates (10^− 3^, 10^− 4^ and 10^− 5^), threshold (Th) (0.3, 0.2 and 0.1) and three types of solvers (sgdm, rmsprop and adam).

### Vehicle detection performance of faster R-CNN with SGDM

**Fig. 15:**
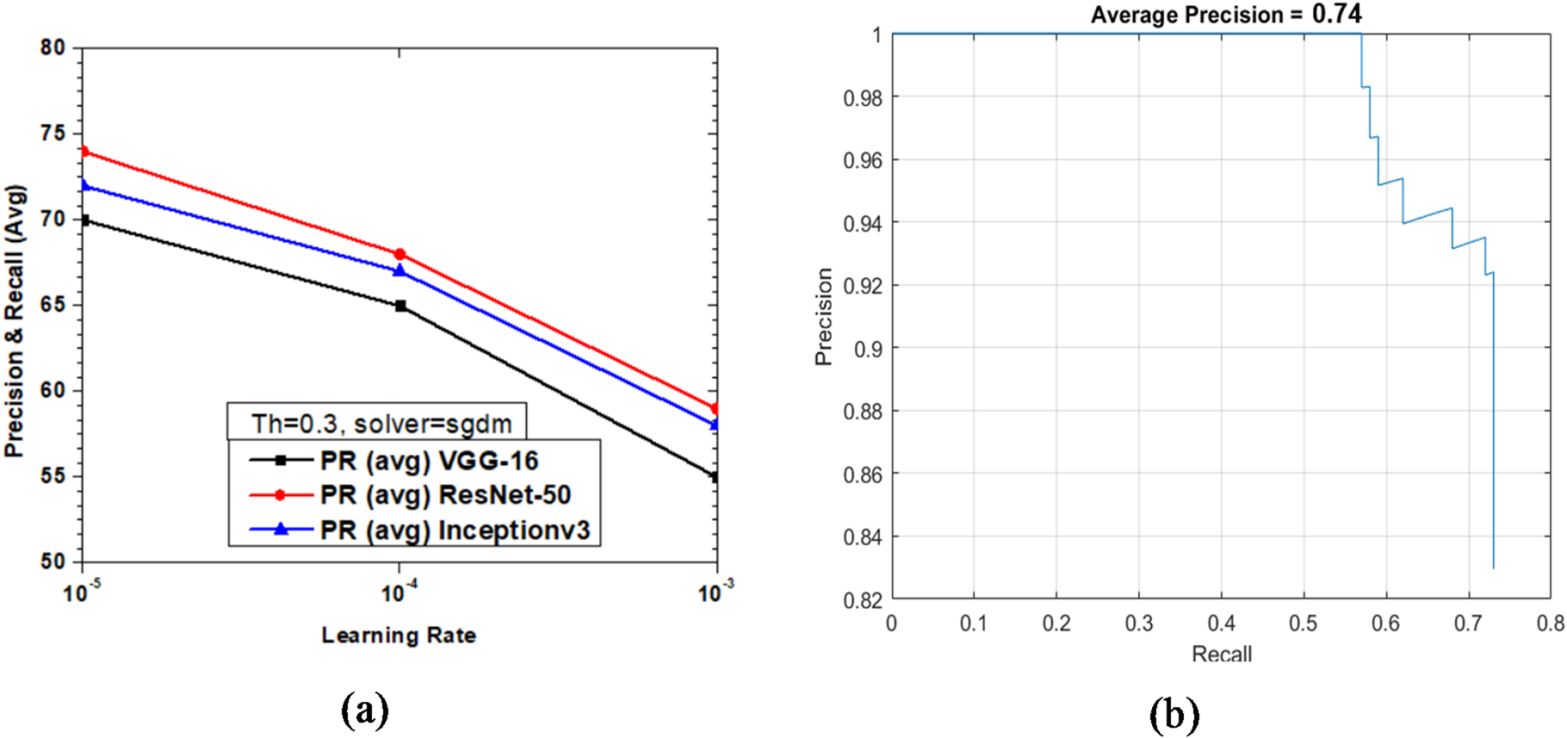
Performance of Faster R-CNN with sgdm solver (Th = 0.3): (**a**) PR (Avg) variations and (**b**) Precision vs. Recall curve for ResNet-50 based network at learning rate = 10^-5^.

Figure 15 shows the variations of average PR value and Precision vs. Recall Curve for Faster R-CNN networks using sgdm solver at detection threshold (Th) = 0.3. The VGG-16, ResNet-50 and Inceptionv3 based Faster R-CNN networks were tested with different solvers (sgdm, rmsprop, adam) and learning rate (10^− 3^, 10^− 4^, 10^− 5^), and the precision recall curves have been obtained for the same. The maximum epoch and mini batch size are set as 10 and 1, respectively.

**Fig. 16:**
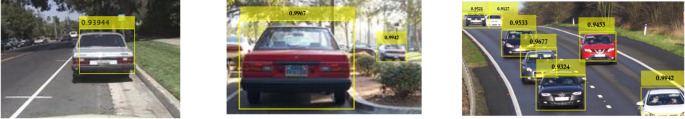
Confidence scores for vehicle detection using ResNet-50 based Faster R-CNN using sgdm solver, Th = 0.3 and learning rate = 10^− 5^.

It was observed that as the learning rate reduced from 10^− 3^ to 10^− 5^ the Precision Recall average (PR (avg)) increased for VGG-16, ResNet-50 and Inception-v3. However, it must be noted that Faster R-CNN based on ResNet-50 performed the best with the PR (avg) value of 74% (learning rate = 10^− 5^) followed closely by 72% of Inceptionv3 at the same learning rate. This clearly shows that there is an optimized value of learning rate solver type which may have a positive or detrimental effect for arriving at the best possible network configuration for vehicle detection. It is observed that VGG-16 achieved only a max PR (avg) value of 70% at learning rate = 10^− 5^ as shown in Fig. 15(a). Figure 15(b) shows the Precision vs. Recall curve for the optimized combination of ResNet-50 based Faster R-CNN with sgdm solver at learning rate = 10^− 5^. Representative examples of detection scores are shown for the corresponding optimized network in Fig. 16.

**Fig. 17:**
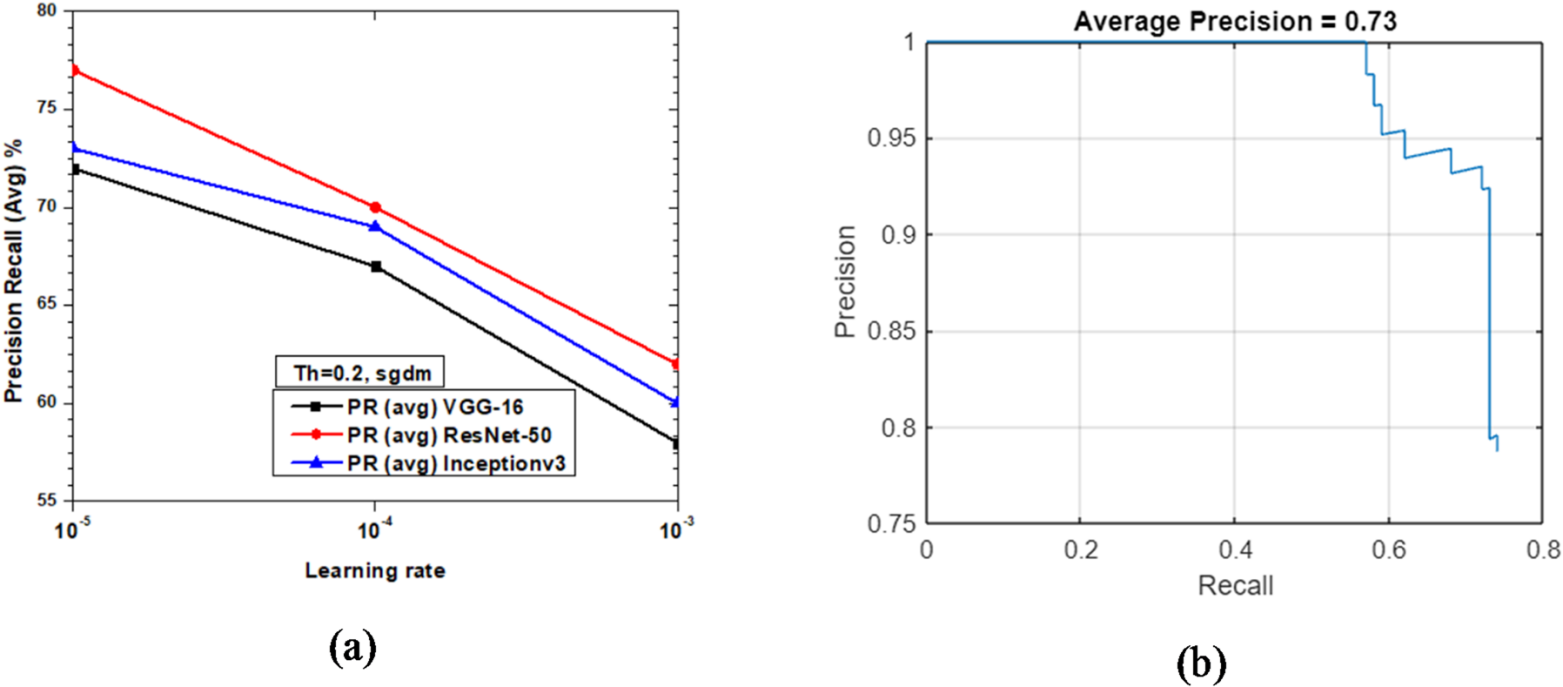
Performance of Faster R-CNN with sgdm solver (Th = 0.2): (**a**) PR (Avg) variations and (**b**) Precision vs. Recall curve for ResNet-50 based network at learning rate = 10^-5^.

**Fig. 18:**
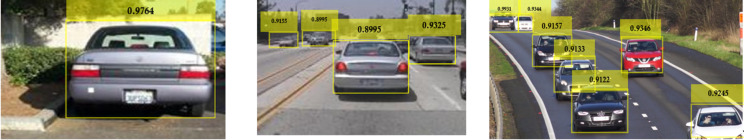
Confidence scores for vehicle detection using ResNet-50 based Faster R-CNN using sgdm solver, Th = 0.2 and learning rate = 10^− 5^.

Figure 17 shows the variations of average PR value and Precision vs. Recall Curve for Faster R-CNN networks using sgdm solver at detection threshold (Th) = 0.2. It was observed that at learning rate 10^− 3^ the PR (avg) for ResNet-50 is 62% which is better in comparison to Inceptionv3 and VGG-16 based networks. It is interesting to note that at learning rate 10^− 4^, the PR (avg) of ResNet-50 and Inceptionv3 are 70 and 69%, respectively. However, it is imperative to observe that the best PR (avg) is 77% achieved by ResNet-50 at learning rate = 10^− 5^ and lowest PR (avg) is 72% by VGG-16 at the same learning rate. Inceptionv3 performs slightly better than VGG-16 with PR (avg) of 73% (refer Fig. 17(a)). Figure 17(b) shows the Precision vs. Recall curve for the optimized combination of ResNet-50 based Faster R-CNN with sgdm solver at learning rate = 10^− 5^. Representative examples of detection scores are shown for the corresponding optimized network in Fig. 18.

**Fig. 19:**
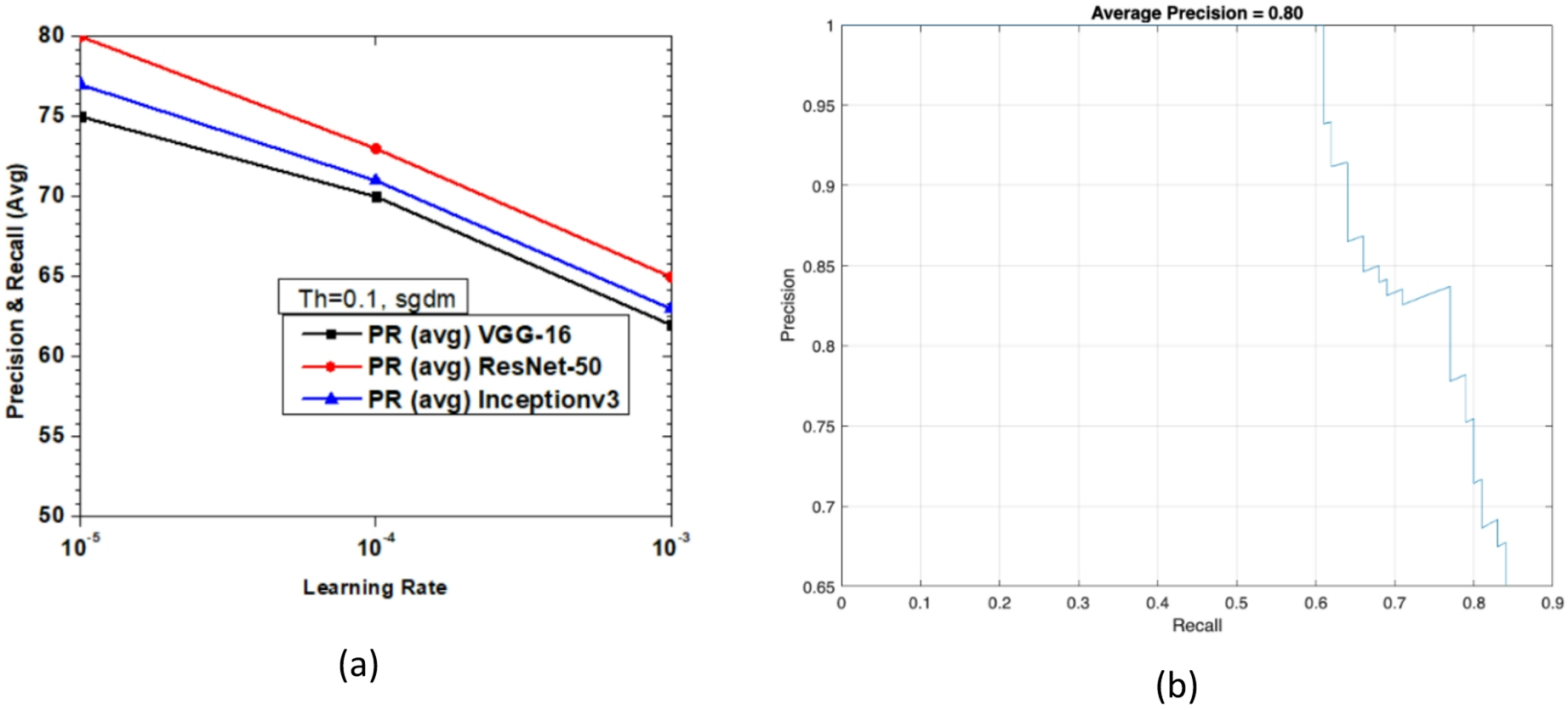
Performance of Faster R-CNN with sgdm solver (Th = 0.1): (**a**) PR (Avg) variations and (**b**) Precision vs. Recall curve for ResNet-50 based network at learning rate = 10^− 5^.

**Fig. 20:**
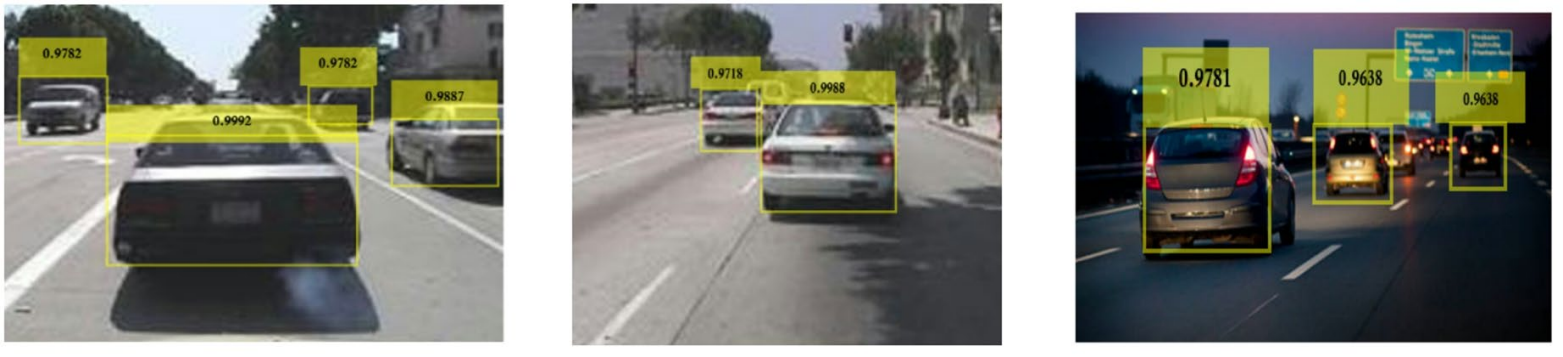
Confidence scores for vehicle detection using ResNet-50 based Faster R-CNN using sgdm solver, Th = 0.1 and learning rate = 10^− 5^.

Figure 19 shows the variations of average PR value and Precision vs. Recall Curve for Faster R-CNN networks using sgdm solver at detection threshold (Th) = 0.1. It is observed that for learning rates between 10^− 3^ and 10^− 5^, the maximum value of PR (avg) obtained is 80% at a learning rate of 10^− 5^ for ResNet-50 as seen from Fig. 19(a). But at the same learning rate the maximum value of PR (avg) of Inceptionv3 and VGG-16 were very close to each other at 77% and 75%, respectively. It is important to note the network performance of VGG-16 and Inceptionv3 is very close at learning rate 10^− 4^ as seen in Fig. 19(a). Figure 19(b) shows the Precision vs. Recall curve for the optimized combination of ResNet-50 based Faster R-CNN with sgdm solver at learning rate = 10^− 5^. Representative examples of detection scores are shown for the corresponding optimized network in Fig. 20.

### Vehicle detection performance of faster R-CNN with RMSPROP

**Fig. 21:**
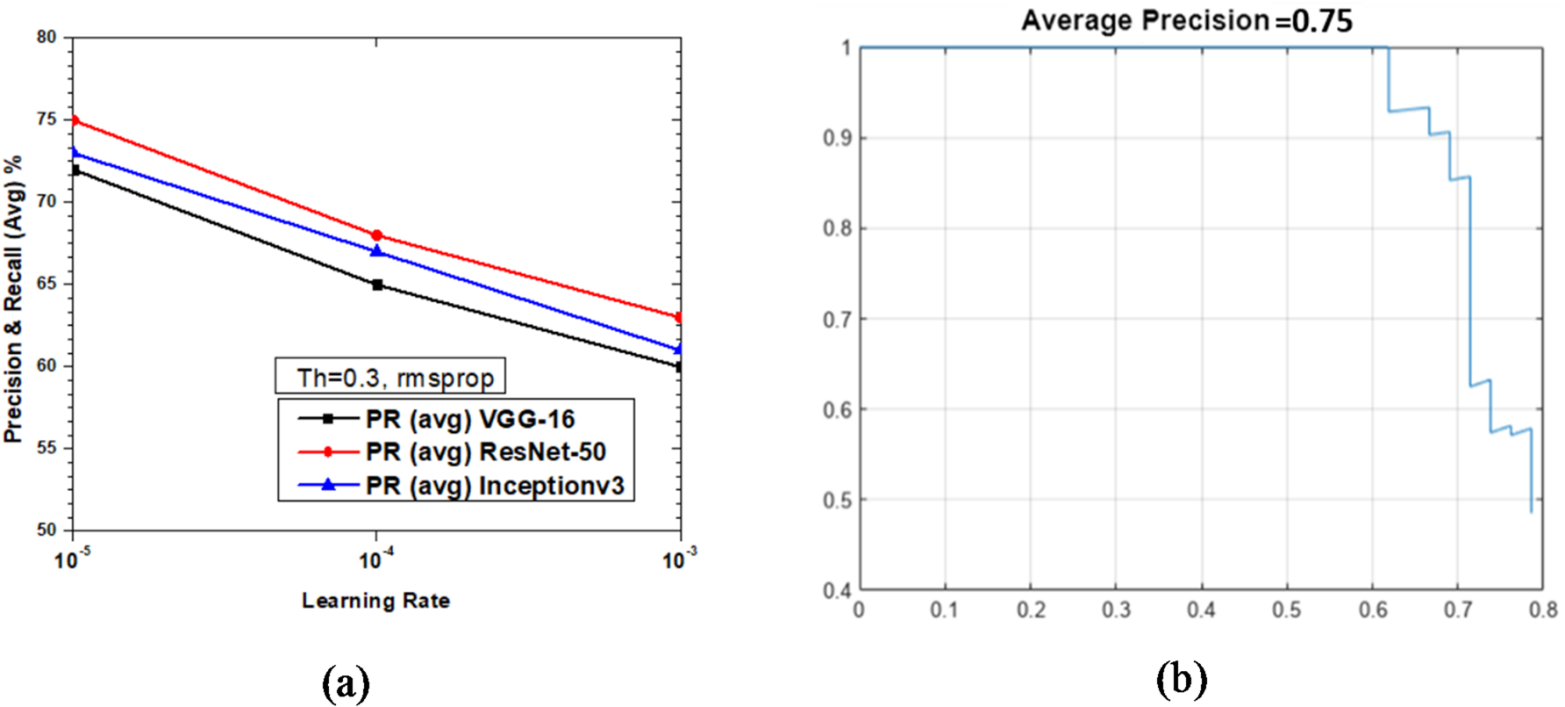
Performance of Faster R-CNN with rmsprop solver (Th = 0.3): (**a**) PR (Avg) variations and (**b**) Precision vs. Recall curve for ResNet-50 based network at learning rate = 10^-5^.

**Fig. 22:**
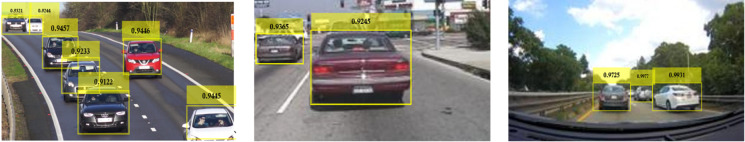
Confidence scores for vehicle detection using ResNet-50 based Faster R-CNN using rmsprop solver, Th = 0.3 and learning rate = 10^− 5^.

Figure 21 shows the variations of average PR value and Precision vs. Recall Curve for Faster R-CNN networks using rmsprop solver at detection threshold (Th) = 0.3. It is observed that as learning rate varies from 10^− 3^ to 10^− 5^, the PR (avg) increased for VGG-16, ResNet-50 and Inceptionv3. It is seen that at learning rate = 10^− 3^ the network performance of VGG-16, ResNet-50 and Inceptionv3 are close to each other with PR avg value of 60–63% which clearly shows that it is not the optimal performance. However, it must be noted that Faster R-CNN based on ResNet-50 attained the best PR (avg) value of 75% followed closely by Inceptionv3 and VGG-16 with 73% and 72%, respectively as seen in Fig. 21(a). Figure 21(b) shows the Precision vs. Recall curve for the optimized combination of ResNet-50 based Faster R-CNN with rmsprop solver at learning rate = 10^− 5^. Representative examples of detection scores are shown for the corresponding optimized network in Fig. 22.

**Fig. 23:**
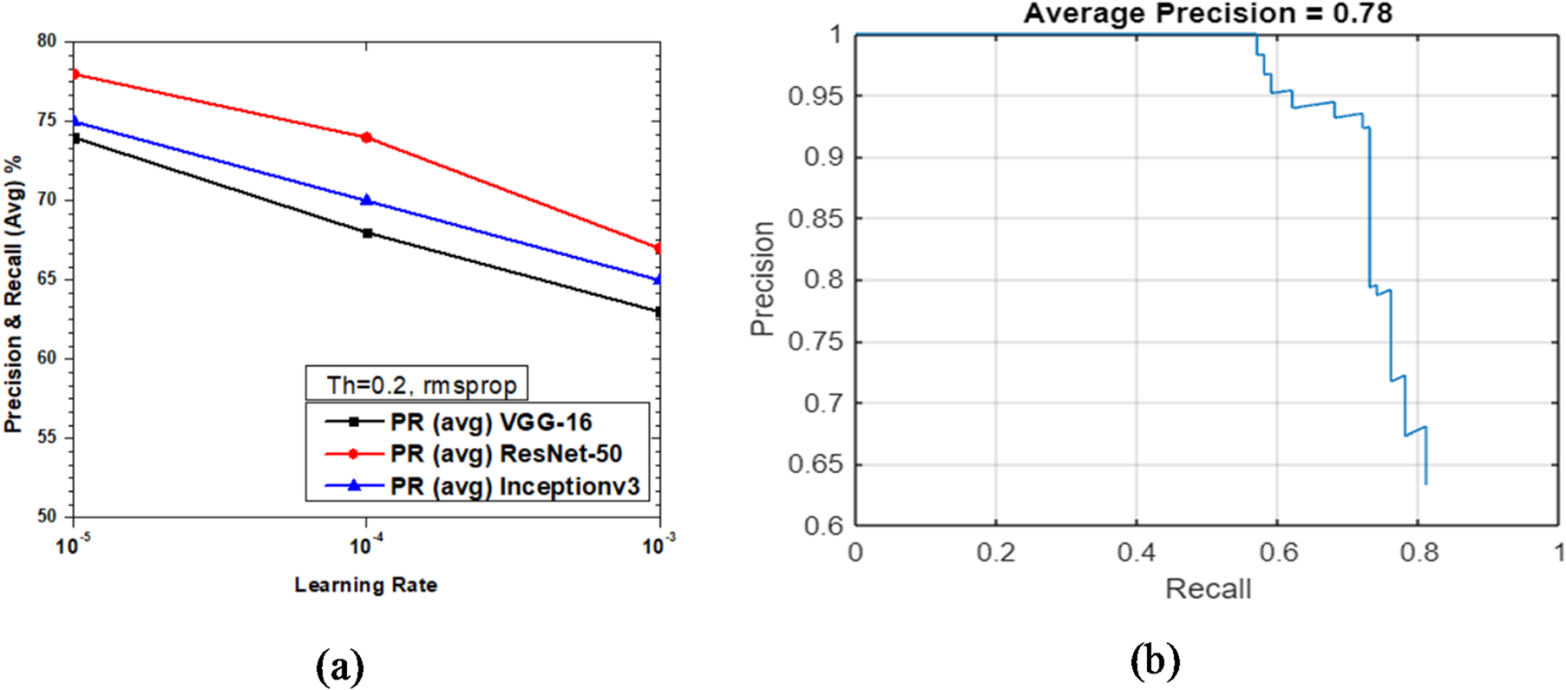
Performance of Faster R-CNN with rmsprop solver (Th = 0.2): (**a**) PR (Avg) variations and (**b**) Precision vs. Recall curve for ResNet-50 based network at learning rate = 10^-5^.

**Fig. 24:**
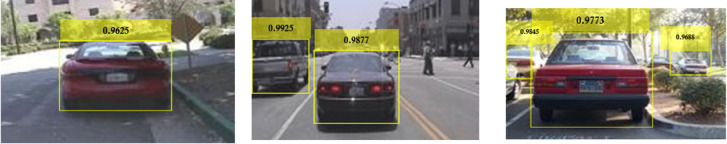
Confidence scores for vehicle detection using ResNet-50 based Faster R-CNN using rmsprop solver, Th = 0.2 and learning rate = 10^− 5^.

Figure 23 shows the variations of average PR value and Precision vs. Recall Curve for Faster R-CNN networks using rmsprop solver at detection threshold (Th) = 0.2. It was observed that as learning rates varies between 10^− 3^ to 10^− 5^, the maximum value of PR (avg) obtained was 78% (at learning rate = 10^− 5^) for ResNet-50 as seen from Fig. 23(a), but at the same learning rate the maximum PR (avg) of Inceptionv3 and VGG-16 were very close to each other, at 75% and 74%, respectively, which clearly shows these are the optimized parameters for enhanced network performance. Figure 23(b) shows the Precision vs. Recall curve for the optimized combination of ResNet-50 based Faster R-CNN with rmsprop solver at learning rate = 10^− 5^. Representative examples of detection scores are shown for the corresponding optimized network in Fig. 24.

**Fig. 25:**
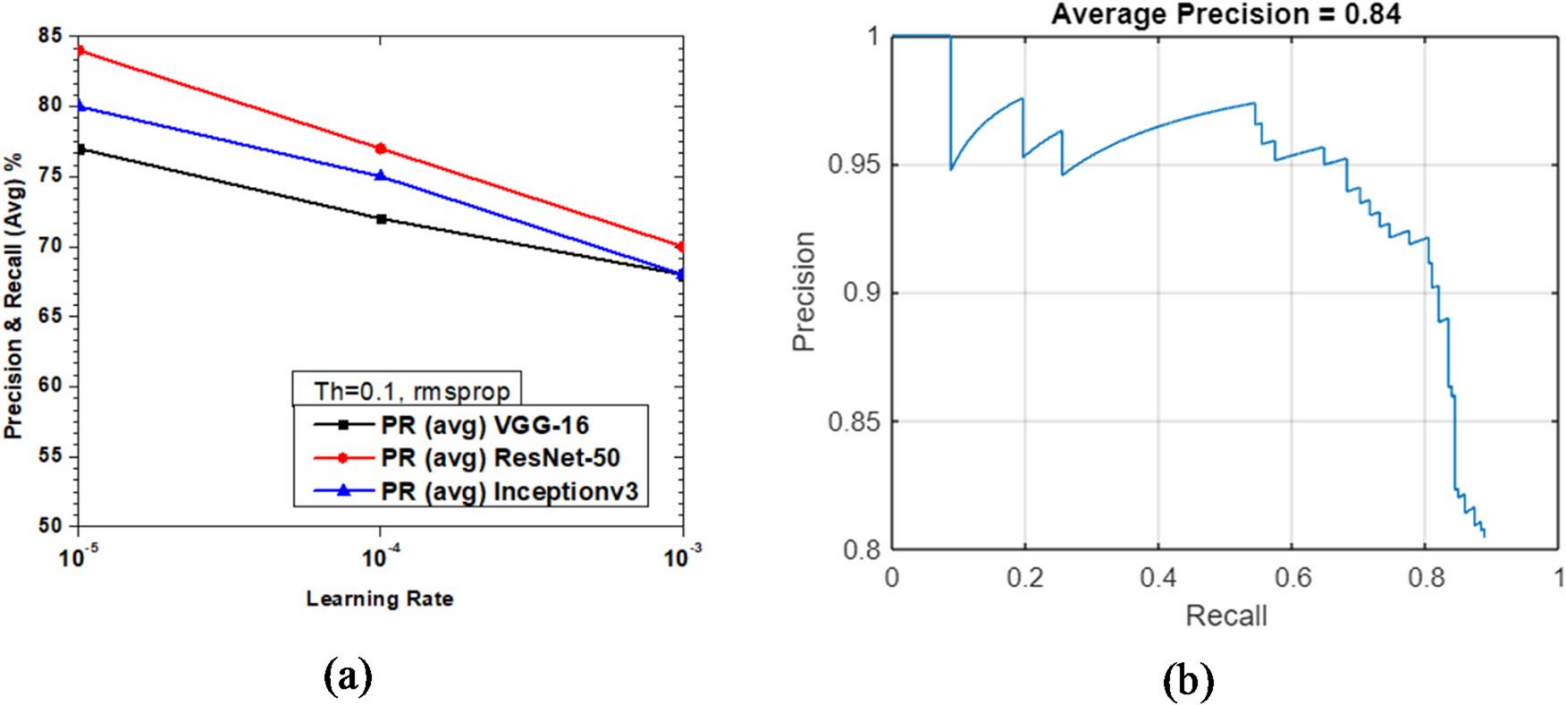
Performance of Faster R-CNN with rmsprop solver (Th = 0.1): (**a**) PR (Avg) variations and (**b**) Precision vs. Recall curve for ResNet-50 based network at learning rate = 10^-5^.

**Fig. 26:**
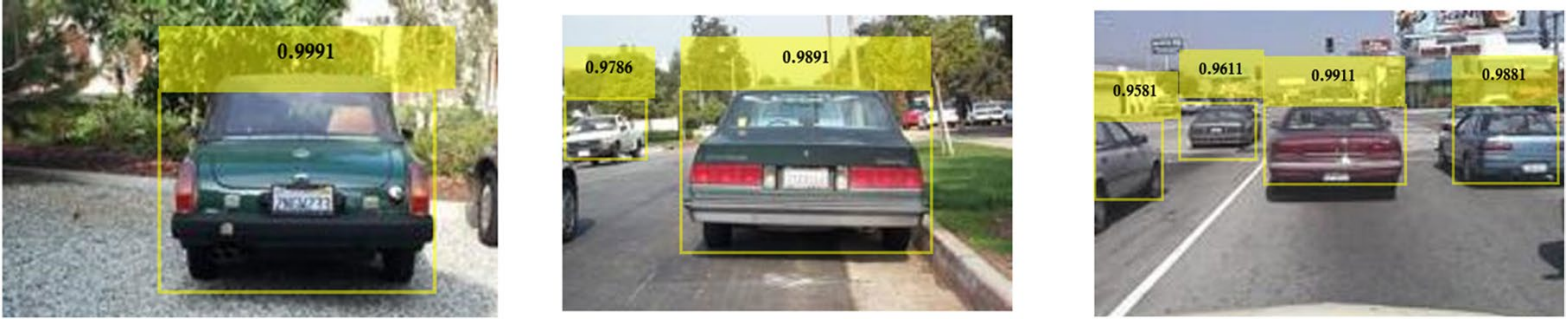
Confidence scores for vehicle detection using ResNet-50 based Faster R-CNN using rmsprop solver, Th = 0.1 and learning rate = 10^− 5^.

Figure 25 shows the variations of average PR value and Precision vs. Recall Curve for Faster R-CNN networks using rmsprop solver at detection threshold (Th) = 0.1. It is observed that as learning rates reduce from 10^− 3^ to 10^− 5^, the maximum value of PR (avg) obtained was 84% (learning rate = 10^− 5^) for ResNet-50 as seen from Fig. 25(a), whereas Inceptionv3 performs slightly lesser than ResNet-50 yielding a PR (avg) of 80% at the same learning rate of 10^− 5^. VGG-16 manages to attain the lowest PR (avg) of 68% at the learning rate of 10^− 3^ as seen in Fig. 25(a). Figure 25(b) shows the Precision vs. Recall curve for the optimized combination of ResNet-50 based Faster R-CNN with rmsprop solver at learning rate = 10^− 5^. Representative examples of detection scores are shown for the corresponding optimized network in Fig. 26.

### Vehicle detection performance of faster R-CNN with ADAM

**Fig. 27:**
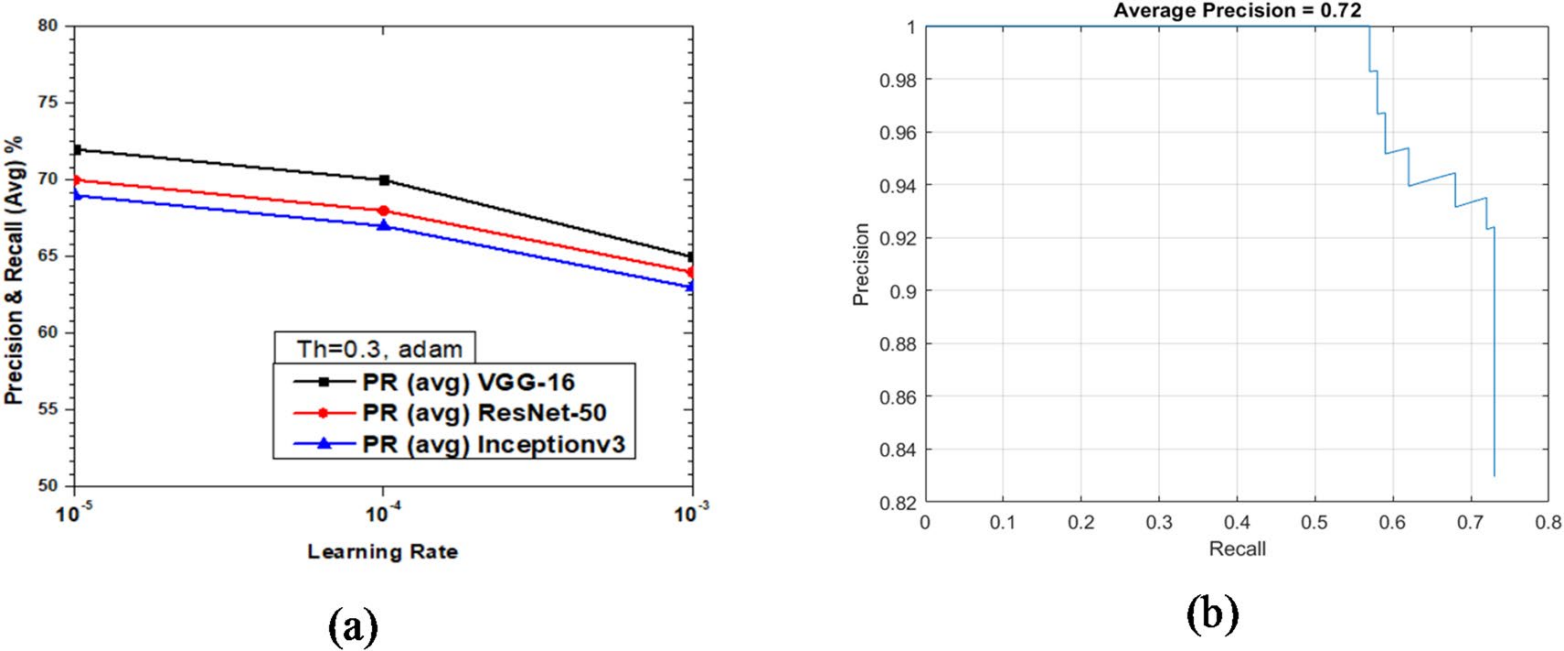
Performance of Faster R-CNN with adam solver (Th = 0.3): (**a**) PR (Avg) variations and (**b**) Precision vs. Recall curve for ResNet-50 based network at learning rate = 10^-5^.

**Fig. 28:**
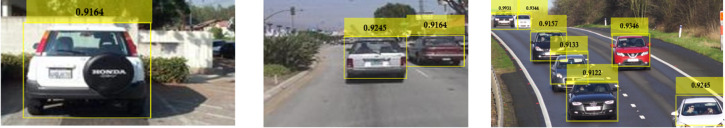
Confidence scores for vehicle detection using ResNet-50 based Faster R-CNN using adam solver, Th = 0.3 and learning rate = 10^− 5^.

Figure 27 shows the variations of average PR value and Precision vs. Recall Curve for Faster R-CNN networks using adam solver at detection threshold (Th) = 0.3. It is observed that as learning rates reduce from 10^− 3^ to 10^− 5^, the PR (avg) for VGG-16 is better than ResNet-50 and Inceptionv3. It is seen from Fig. 27(a) that at learning rate = 10^− 3^ the network performance of Inceptionv3 has the least value PR avg value of 63% as compared to 65% for VGG-16. However, it must be noted that Faster R-CNN based on VGG-16 performed the best with a PR (avg) value of 72% while ResNet-50 and Inceptionv3 yielded 70% and 69%, respectively, as shown in Fig. 27(a). The Precision vs. Recall curve for the optimized combination of ResNet-50 based Faster R-CNN with adam solver at learning rate = 10^− 5^ is shown in Fig. 27(b). Representative examples of detection scores are shown for the corresponding optimized network in Fig. 28.

**Fig. 29:**
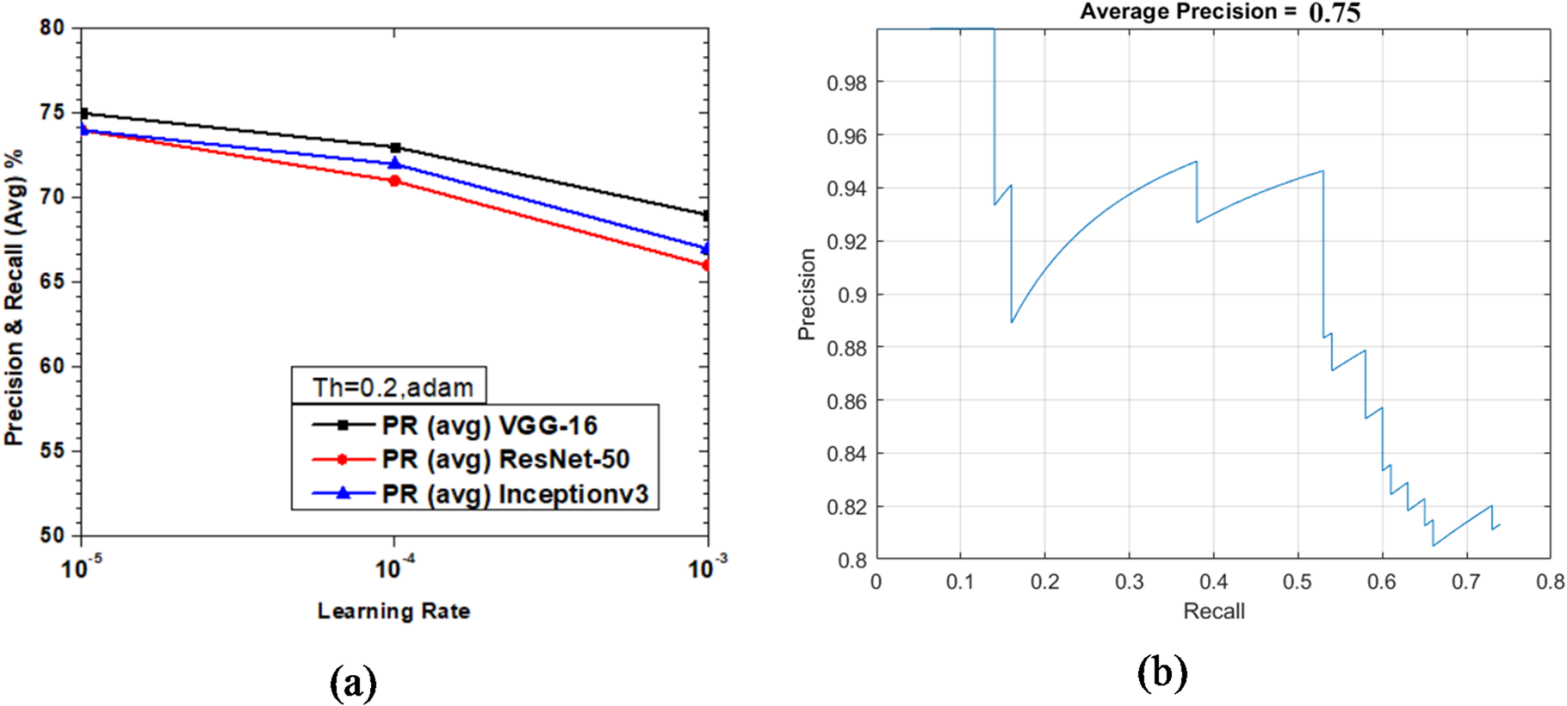
Performance of Faster R-CNN with adam solver (Th = 0.2): (**a**) PR (Avg) variations and (**b**) Precision vs. Recall curve for ResNet-50 based network at learning rate = 10^-5^.

**Fig. 30:**
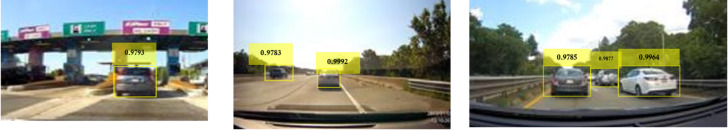
Confidence scores for vehicle detection using ResNet-50 based Faster R-CNN using adam solver, Th = 0.2 and learning rate = 10^− 5^.

Figure 29 shows the variations of average PR value and Precision vs. Recall Curve for Faster R-CNN networks using adam solver at detection threshold (Th) = 0.2. It is observed that with learning rate variations from 10^− 3^ to 10^− 5^, the max value of PR (avg) obtained was 78% (learning rate = 10^− 5^) for ResNet-50 as seen from Fig. 29(a), but at the same learning rate the maximum value of PR (avg) of VGG-16 & Inceptionv3 were very close to each other at 75% and 74%, respectively, which clearly shows these are the optimized parameters for enhanced network performance. The Precision vs. Recall curve for the optimized combination of ResNet-50 based Faster R-CNN with adam solver at learning rate = 10^− 5^ is shown in Fig. 29(b). Representative examples of detection scores are shown for the corresponding optimized network in Fig. 30.

**Fig. 31:**
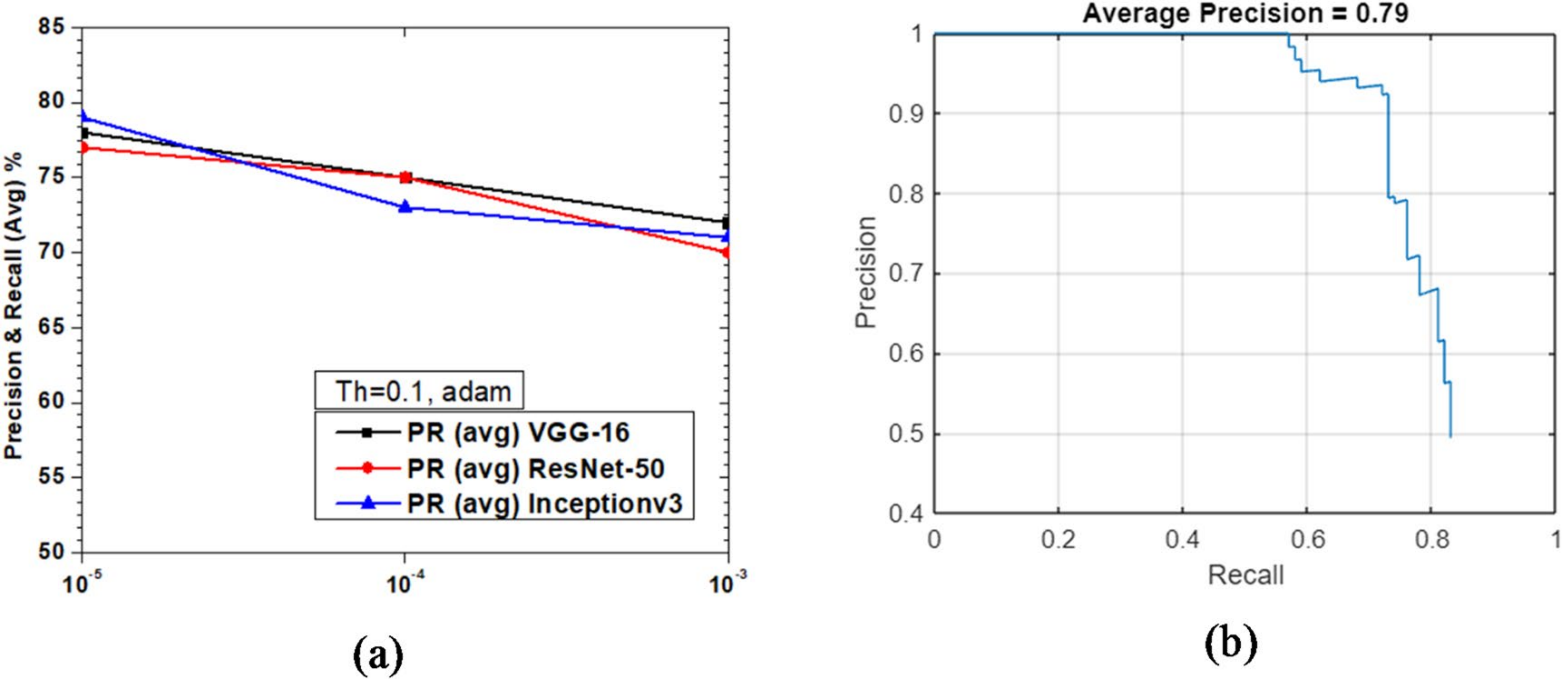
Performance of Faster R-CNN with adam solver (Th = 0.1): (**a**) PR (Avg) variations and (**b**) Precision vs. Recall curve for ResNet-50 based network at learning rate = 10^− 5^.

**Fig. 32:**
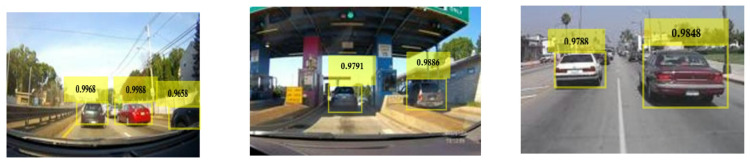
Confidence scores for vehicle detection using ResNet-50 based Faster R-CNN using adam solver, Th = 0.1 and learning rate = 10^− 5^.

Figure 31 shows the variations of average PR value and Precision vs. Recall Curve for Faster R-CNN networks using adam solver at detection threshold (Th) = 0.1. It is observed that with the variation of learning rates between 10^− 3^ and 10^− 5^, the maximum value of PR (avg) obtained was 79% (Th = 0.1, learning rate = 10^− 5^). There are some interesting trends to observe in Fig. 31(a). It is seen that all the network performance is very close to each other, varying about 2–3% at all learning rates from 10^− 3^ to 10^− 5^. This shows that threshold plays a pivotal role in the estimation of network efficiency and the lower the value of threshold the more is the probability of vehicle detection. The Precision vs. Recall curve for the optimized combination of ResNet-50 based Faster R-CNN with adam solver at learning rate = 10^− 5^ is shown in Fig. 31(b). Representative examples of detection scores are shown for the corresponding optimized network in Fig. 32.

Table 2 presents a summary of the parametric analysis conducted in this study. Lower levels of both threshold and learning rates yielded the best results. Among the three base CNN networks considered, ResNet-50 demonstrated the highest performance and rmsprop helped achieve the best results among the three solvers. Overall, the maximum PR (avg) performance of 84% was achieved with the ResNet-50 network using rmsprop solver with a threshold of 0.1 and learning rate of 10^− 5^.

**Table 2:** Optimum hyperparameter combination of faster R-CNN with different base CNN using different solvers.

Base CNN	Solver	Threshold	Learning rate	PR (avg)
Inceptionv3	sgdm	0.1	10^− 5^	77%
	rmsprop	0.1	10^− 5^	80%
	adam	0.1	10^− 5^	78%
ResNet-50	sgdm	0.1	10^− 5^	80%
	rmsprop	0.1	10^− 5^	84%
	adam	0.1	10^− 5^	79%
VGG-16	sgdm	0.1	10^− 5^	75%
	rmsprop	0.1	10^− 5^	77%
	adam	0.1	10^− 5^	77%

## Comparative analysis

Table 3 presents a comparative analysis of existing studies that have improved the performance of Faster R-CNN through various modifications. While prior works have focused on different aspects such as small object scaling^20^, backbone CNN variations^21,23,28^, and comparisons with other object detection algorithms^25,26^, a systematic evaluation of solver type, detection threshold, and base CNN architecture has not been conducted. Notably, the effect of solver type and base CNN selection has introduced significant variations in detection accuracy, with differences of approximately 10% across different models (refer Table 2, Sect. "Vehicle detection performance of faster R-CNN with SGDM" to "Vehicle detection performance of faster R-CNN with ADAM"). This highlights the critical influence of these hyperparameters on model performance. In contrast, the present study systematically investigates these factors, offering a comprehensive analysis of their impact on vehicle detection accuracy. Although the maximum performance achieved (84%) is comparable to prior work, further improvements could be explored by increasing dataset size and incorporating class imbalance handling techniques^42^.

The performance of the three solvers (sgdm, adam, rmsprop) exhibited distinct patterns due to their gradient update mechanisms. Sgdm applies momentum-driven updates that help smooth oscillations but are highly sensitive to the learning rate, converging slowly if the rate is too low and overshooting minima if too high. Adam combines momentum with adaptive learning rates for each parameter, allowing rapid convergence, but in our experiments, it occasionally led to overfitting given the small dataset, as its aggressive parameter updates exploited noise. rmsprop, on the other hand, adaptively scales the learning rate by normalizing with an exponentially decaying average of squared gradients, making it particularly effective for non-stationary and sparse gradient problems such as those in RPN of Faster R-CNN. This adaptive scaling mitigated instability during training and accounted for varying gradient magnitudes across layers, which explains why rmsprop consistently outperformed sgdm and adam across all learning rates and detection thresholds. Overall, the results indicate that solver choice interacts strongly with learning rate and dataset size, and the superior performance of rmsprop underscores the importance of adaptive gradient normalization in object detection tasks where region-based learning is central.

As illustrated in Figs. 15, 17 and 19 (sgdm), Figs. 21, 23 and 25 (rmsprop), and Figs. 27, 29 and 31 (adam), the detection threshold significantly influences the balance between precision and recall. At a higher threshold (0.3), the model achieves greater precision by reducing false positives but at the cost of lower recall, often missing smaller or partially occluded vehicles. Conversely, at a lower threshold (0.1), recall improves considerably as more true positives are identified, though at the expense of increased false positives. These results confirm the well-known trade-off between precision and recall, and demonstrate that threshold selection should be scenario-dependent: for example, a lower threshold may be preferable in safety-critical applications such as autonomous driving, whereas a higher threshold may be suitable for traffic surveillance where minimizing false alarms is more critical.

**Table 3:** Performance analysis of different studies using faster R-CNN and other state-of-the-art methods in literature in comparison with present work.

Reference	Method used	Maximum PR (Avg)	Limitations
^20^	Scaling small-sized objects (Faster R-CNN)	82%	Influence of solver type, threshold and base CNN not evaluated.
^21^	Faster R-CNN with different base CNNs: VGG-16, MobileNetv2, ResNet-50, ResNet-101	83%	Influence of solver type and threshold not evaluated.
^23^	Modified ResNet model as backbone CNN (Faster R-CNN)	83%	Influence of solver type and threshold not evaluated.
^25^	Faster R-CNN, YOLOv3, and Single Shot MultiBox Detector (SSD)	85%	Influence of solver type, threshold and base CNN not evaluated.
^26^	Faster R-CNN, SSD (UAV-based detection)	81%	Influence of solver type, threshold and base CNN not evaluated.
^28^	Faster R-CNN with Inceptionv2, ResNet-101	91%	Influence of solver type and threshold not evaluated.
YOLOv5^43^, YOLOv8^44^	YOLO family (real-time object detectors)	86–92%	Faster but often with reduced accuracy on small/occluded vehicles.
Present study	Faster R-CNN with ResNet-50 + rmsprop, Th = 0.1, LR = 10^− 5^	84%	Dataset size relatively small; occlusion handling can be improved.

Our study aligns with ongoing efforts to enhance perception systems in autonomous driving. Zhuang et al.^45^ tackled the challenge of novel object detection using a few-shot learning approach, whereas we focus on optimizing the classical Faster R-CNN framework through hyperparameter tuning to improve accuracy and inference efficiency for general vehicle detection. Similarly, Mohammadian et al.^46^ examined edge-aware traffic monitoring by integrating lightweight CNNs and attention mechanisms, emphasizing deployment on resource-constrained platforms. Our work shares this objective of computational efficiency but employs a different detection backbone and optimization strategy. Finally, Wang et al.^47^ explored multi-agent imitation learning to simulate social driving interactions, which builds upon accurate perception inputs such as the ones provided by our improved detection model. Together, these studies reflect a shared goal of building robust, efficient, and scalable intelligent transportation systems.

## System requirements for Real-Time implementation

Implementing the Faster R-CNN model for vehicle detection in a real-time system, such as an autonomous vehicle or intelligent traffic monitoring setup, requires both adequate hardware and optimization strategies. MATLAB provides native support for deep learning deployment, and when combined with hardware acceleration (GPUs or embedded AI platforms), the proposed model can achieve near real-time detection.

For embedded deployment, devices such as the NVIDIA Jetson Xavier NX or Jetson AGX Xavier are suitable, as they balance compact form factors with high-performance AI inference capability. The Jetson Xavier NX can typically achieve 20–30 fps with an optimized Faster R-CNN, while the Jetson AGX Xavier can process high-resolution inputs (1080p/4K) at even higher frame rates. On traditional platforms, Intel Core i7/i9 CPUs combined with CUDA-enabled GPUs (e.g., RTX 2080 Ti or later) are appropriate, with at least 8 GB RAM and SSD storage for efficient data handling.

To reduce inference time and computational load while maintaining accuracy, several optimization strategies can be employed: (i) model compression via pruning and quantization to reduce parameter count, (ii) batch size tuning to optimize throughput and latency balance, (iii) knowledge distillation where the optimized Faster R-CNN serves as a teacher to train a lighter student network for deployment, and (iv) mixed-precision inference to accelerate computation on GPUs. These strategies collectively enable the proposed framework to operate efficiently on embedded devices without substantial loss of detection accuracy.

## Limitations and future work

The size of the data set used in the current work is still minimal. It could be further enhanced by finding more data and applying various data augmentation techniques (e.g., zoom/crop, and rotate) to increase the data size. Recent works have explored the Pixel-wise Adaptive Training^46^ and Position-Aware Transformer^47^ for object detection problems with high levels of class imbalance. Such approaches can be adopted in the future to improve the robustness of the model predictions.

Although our study employed constant learning rates for systematic grid search, alternative strategies such as step decay, exponential decay, and cyclical learning rates have been shown in literature to improve convergence speed and model robustness. Future work will explore dynamic learning rate schedules such as cyclical learning rates and exponential decay, which may enhance convergence stability and efficiency compared to fixed learning rates. These methods could help the Faster R-CNN model achieve improved accuracy with faster training convergence in vehicle detection applications^48–50^.

The present work is limited by the relatively small dataset size, which may restrict the robustness of the trained models in diverse real-world conditions. Although 5-fold cross-validation was employed to reduce overfitting and provide a more reliable estimate of model generalization, a larger and more diverse datasets such as, KITTI, BDD100K, and UA-DETRAC, would likely improve robustness, particularly in rare scenarios such as night-time driving, adverse weather, and heavy occlusions. Performance on small and heavily occluded vehicles was observed to be lower, consistent with findings in related studies. While anchor boxes partially address scale variance, the Faster R-CNN framework in its current form struggles with occlusion-rich traffic scenes.

Another limitation is the absence of formal statistical significance testing due to dataset constraints. While precision–recall curves across thresholds and folds (Figs. 15, 16, 17, 18, 19, 20, 21, 22, 23, 24, 25, 26, 27, 28, 29, 30 and 31) illustrate consistency of results, future studies with larger datasets will incorporate statistical validation methods (e.g., confidence intervals, hypothesis testing) to rigorously substantiate observed performance differences between solvers, learning rates, and detection thresholds.

Future work will address these limitations in several ways: (i) expanding the dataset to include more diverse environmental conditions (urban vs. rural, day vs. night, varied weather), (ii) incorporating advanced data augmentation techniques, including occlusion-aware strategies such as Cutout, Mixup, and Copy-Paste, (iii) leveraging synthetic datasets generated from simulators such as CARLA or Unity, as well as augmentation-based synthetic approaches such as GAN-based generation, to provide additional variability without costly manual annotation^49–52^, (iv) integrating multi-scale feature extraction modules such as Feature Pyramid Networks (FPN) to improve small-object detection, (v) experimenting with dynamic learning rate schedules (e.g., cyclical or exponential decay) to improve training stability, and (vi) extending the framework toward multimodal fusion (e.g., lidar, radar, or infrared) for robust detection under low-visibility conditions^52,53^. In addition, optimization strategies such as pruning, quantization, and knowledge distillation will be explored to reduce inference time for real-time deployment on embedded systems. Together, these directions will enhance both the accuracy and the practical applicability of the proposed framework in real-world intelligent transportation systems.

## Conclusions

The present work demonstrated a systematic approach for optimizing the important hyperparameters of object detection models, particularly in the case of vehicle detection tasks. The Faster R-CNN model was optimized for vehicle detection activity by assessing the effects of type of base CNN (VGG-16, ResNet-50, Inceptionv3), type of solver (sgdm, rmsprop, adam), learning rate (10^− 5^, 10^− 4^, 10^− 3^) and detection threshold (0.1, 0.2, 0.3) on its performance. The maximum value obtained of PR (avg) is 84% using ResNet-50 at a learning rate = 10^− 5^, Th = 0.1, using rmsprop solver for the entire range of learning rates and detection threshold considered in this study. It is clearly seen that as the learning rates reduce from 10^− 3^ to 10^− 5^, it causes the network efficiency (PR avg) to rise in a steady manner. However, it must be noted the solver type also significantly impacts network efficiency. The threshold value set for the network during this process also governs the overall performance of the network as seen from the results. Thus, it is important for the object detection algorithm to incorporate the best optimized values of network type, learning rate, solver type, and threshold value to extract maximum efficiency in object detection tasks.

## Data Availability

The data that support the findings of this study are available from the corresponding author upon reasonable request.
